# Synthesis of benzoxazoles *via* an iron-catalyzed domino C–N/C–O cross-coupling reaction†

**DOI:** 10.1039/c7ra13080e

**Published:** 2018-01-09

**Authors:** Bo Yang, Weiye Hu, Songlin Zhang

**Affiliations:** Key Laboratory of Organic Synthesis of Jiangsu Province, College of Chemistry, Chemical Engineering and Materials Science, Soochow University Suzhou 215123 P. R. China zhangsl@suda.edu.cn

## Abstract

An eco-friendly and efficient method has been developed for the synthesis of 2-arylbenzoxazoles *via* a domino iron-catalyzed C–N/C–O cross-coupling reaction. Some of the issues typically encountered during the synthesis of 2-arylbenzoxazoles in the presence of palladium and copper catalysts, including poor substrate scope and long reaction times have been addressed using this newly developed iron-catalyzed method.

## Abstract 2

The synthesis of benzoxazoles *via* an iron-catalyzed cascade C–N and C–O coupling is described.

## Graphical abstract

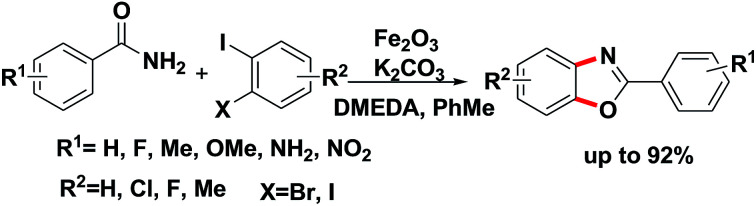

2-Arylbenzoxazoles are an important class of structures in natural products, and pharmaceuticals and has shown a wide range of biological activities, such as antitumor, antiviral, and antimicrobial activities.^1^ In particular, they show a marvellous efficacy in the treatment of duchenne muscular dystrophy (DMD) which is one of the most common of the muscular dystrophies that is caused by a mutation in the gene DMD, located in humans on the X chromosome (Xp21).^2^ So the synthesis of 2-arylbenzoxazoles has been intensively studied for use in organic and medicinal chemistry over the past few years.

Numerous methods have been reported to synthesise this motif, one of the common methods is transition-metal-catalyzed (like Pd,^3^ Ni,^4^ Cu,^5^ Mn^6^*etc.*) cross-coupling from pre-existing benzoxazoles with aryl halide or arylboronic acid. And another method is the classic one employing a cyclocondensation approach between an aminophenol and either a carboxylic acid^7^ or benzaldehyde^8^ (Scheme 1, path a). In 2004, Frank Glorius' group reported a domino copper-catalyzed C–N and C–O cross-coupling for the conversion of primary amides into benzoxazoles^9^ (Scheme 1, path b) which is a new reaction type for the synthesis of benzoxazoles. Bunch *et al.* apply this domino reaction in the synthesis of planar heterocycles in 2014.^10^ In addition the cyclization of *o*-halobenzenamides to benzoxazoles has been reported several times.^11,12^ Nevertheless, some limitations in the reported methods need to be overcome, such as the use of palladium complexes and narrow substrate range.

**Scheme 1:**
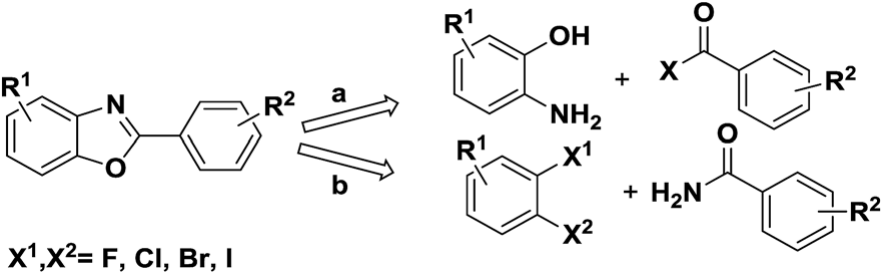
Classic method of benzoxazole formation.

In the last few years, there has been a significant increase in the number of reports pertaining to the development of iron-catalyzed reactions in organic synthesis, where iron has shown several significant advantages over other metals, such as being more abundant, commercially inexpensive, environmentally friendly and drug safety.^13^ Compared with palladium and copper, the use of iron is particularly suitable for reactions involving the preparation of therapeutic agents for human consumption. With this in mind, it was envisaged that an new method should be developed for the synthesis of benzoxazoles *via* an iron-catalyzed domino C–N/C–O cross-coupling reaction.

The reaction of benzamide (1a) with 1-bromo-2-iodobenzene was used as model transformation to identify the optimum reaction conditions by screening a variety of different iron salts, bases, ligands and solvents (Table 1). Several iron salts were screened in this reaction, including FeCl_3_, FeCl_2_·4H_2_O, FeSO_4_·7H_2_O, Fe(acac)_3_, Fe_2_O_3_, Fe_3_O_4_, Fe_3_O_4_(nano), Fe_2_O_3_(nano), Fe_2_(SO_4_)_3_ and Fe(NO_3_)_3_·9H_2_O, Fe_2_O_3_ was found to give the best results with the desired product 3a being formed in a yield of 15% while most of the iron salt have no effect on the reaction (Table 1, entries 1–10). Then, several other bases, including LiO^*t*^Bu, Na_2_CO_3_, NaOAc, KOH and K_2_CO_3_ were also evaluated under the same conditions using Fe_2_O_3_, but all of them failed to provided the desired product 3a except K_2_CO_3_ with a yield of 37% (Table 1, entries 11–15). When the reaction was stirred for 24 h at 110 °C in the presence of 20% mol of Fe_2_O_3_, 20% mol *N*,*N*′-dimethylethanediamine (DMEDA) and 1 equiv. of K_2_CO_3_ in PhMe under nitrogen, (*N*-(2-bromophenyl)benzamide) was obtained as an intermediate which could be converted to the final product with a yield of 87% if extend the reaction time from 24 h to 48 h (Table 1, entries 15, 16). Several ligands were also screened in the model reaction, and the results revealed that the nature of the ligand has a dramatic impact on the yield of the reaction. For example, the use of DMEDA gave 2-phenylbenzo[*d*]oxazole in 85% yield, whereas 1,10-phenanthroline, dipyridyl and l-proline provided no product (Table 1, entries 16–19). The reaction was conducted in DMSO, DMF and PhMe_2_ respectively and none of them provided a much higher of the desired product than toluene (Table 1, entries 20–22). Control experiments was taken in the absence of Fe_2_O_3_, no product was obtained (Table 1, entry 23). In view of the fact that the trace metals in catalytic, as is well-known, sometime could play an important role in the reaction,^14^ high-purity Fe_2_O_3_ (99.999%) and K_2_CO_3_ (99.999%) were applied in the reaction (Table 1, entry 24). The product was formed in a yield of 86% which was similar with the one of the entry 16.

**Table 1:** Optimization of reaction conditions^a^

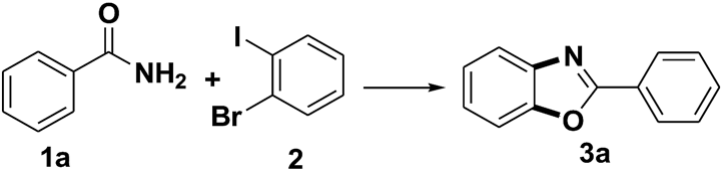					
Entry	Iron salt	Ligand	Base	Solvent	*Y* b (%)
1	FeCl_3_	DMEDA	KO^*t*^Bu	PhMe	Trace
2	FeCl_2_·4H_2_O	DMEDA	KO^*t*^Bu	PhMe	Trace
3	FeSO_4_·7H_2_O	DMEDA	KO^*t*^Bu	PhMe	0
4	Fe(acac)_3_	DMEDA	KO^*t*^Bu	PhMe	0
5	Fe_2_O_3_	DMEDA	KO^*t*^Bu	PhMe	15
6	Fe_3_O_4_	DMEDA	KO^*t*^Bu	PhMe	0
7	Fe_3_O_4_(nano)	DMEDA	KO^*t*^Bu	PhMe	10
8	Fe_2_O_3_(nano)	DMEDA	KO^*t*^Bu	PhMe	0
9	Fe_2_(SO_4_)_3_	DMEDA	KO^*t*^Bu	PhMe	0
10	Fe(NO_3_)_3_·9H_2_O	DMEDA	KO^*t*^Bu	PhMe	0
11	Fe_2_O_3_	DMEDA	LiO^*t*^Bu	PhMe	0
12	Fe_2_O_3_	DMEDA	Na_2_CO_3_	PhMe	0
13	Fe_2_O_3_	DMEDA	NaOAc	PhMe	0
14	Fe_2_O_3_	DMEDA	KOH	PhMe	0
15	Fe_2_O_3_	DMEDA	K_2_CO_3_ (24 h)	PhMe	37
16	Fe_2_O_3_	DMEDA	K_2_CO_3_ (48 h)	PhMe	87
17	Fe_2_O_3_	Phen	K_2_CO_3_	PhMe	Trace
18	Fe_2_O_3_	l-Proline	K_2_CO_3_	PhMe	0
19	Fe_2_O_3_	Dpy	K_2_CO_3_	PhMe	0
20	Fe_2_O_3_	DMEDA	K_2_CO_3_	DMSO	0
21	Fe_2_O_3_	DMEDA	K_2_CO_3_	DMF	0
22	Fe_2_O_3_	DMEDA	K_2_CO_3_	PhMe_2_	0
23	—	DMEDA	K_2_CO_3_	PhMe	0
24	Fe_2_O_3_	DMEDA	K_2_CO_3_	PhMe	86^c^
25	Fe_2_O_3_	DMEDA	K_2_CO_3_	PhMe	58^d^

a Reaction conditions: benzamides (0.5 mmol), 1-bromo-2-iodobenzene (1.5 eq.), iron salt (20% mol), base (1 eq.), ligand (20%) were added to a solvent (2 mL) and react at 110 °C for 48 h under N_2_.

b Isolated yield based on 1a after silica gel chromatography.

c Fe_2_O_3_ and K_2_CO_3_ were applied in purity of 99.999% from alfa.

d with Fe_2_O_3_ in a dosage of 10 mmol%.

At last, the dosage of Fe_2_O_3_ was reduce to 10 mmol%, but only 58% yield was obtained (Table 1, entry 26). Taken together, the results of these screening experiments revealed that the optimal conditions for the reaction were Fe_2_O_3_ (20 mol%), DMEDA (20 mol%) and K_2_CO_3_ (1 eq.) in toluene at 110 °C for 48 h.

It is noteworthy that the intermediate product 4a was formed under the optimized conditions *via* the C–N cross coupling reaction of benzamide (1a) with 1-bromo-2-iodobenzene (2). So a possible pathway of the reaction was proposed as shown in Scheme 2.

**Scheme 2:**
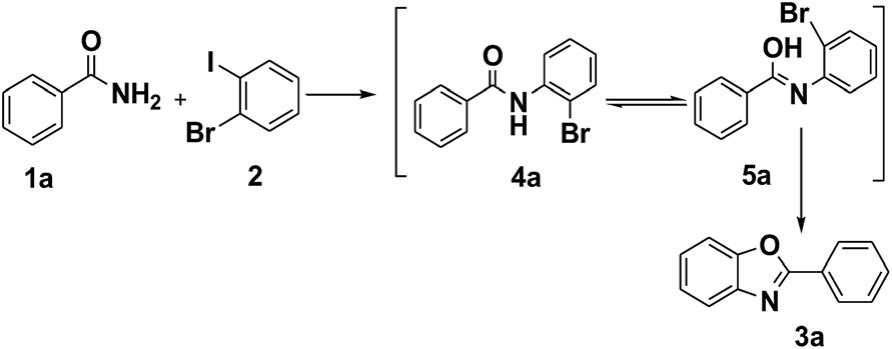
The pathway of the reaction.

With the optimized reaction conditions in hand, we proceeded to investigate the substrate scope of the reaction using a variety of different 1,2-dihalobenzene substrates and aryl formamide (Table 2). Benzamide containing electron poor (3d–f, 3l), electron-neutral (3a–c, 3k), and electron-rich (3g–j, 3m–n) substituents were all obtained in moderate to excellent yields. But some functional groups are intolerated in the reaction, like amino (3p) and nitro (3q).

**Table 2:** Reagent scope of reaction^a^

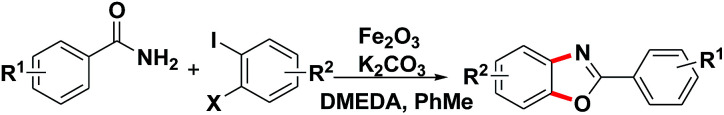
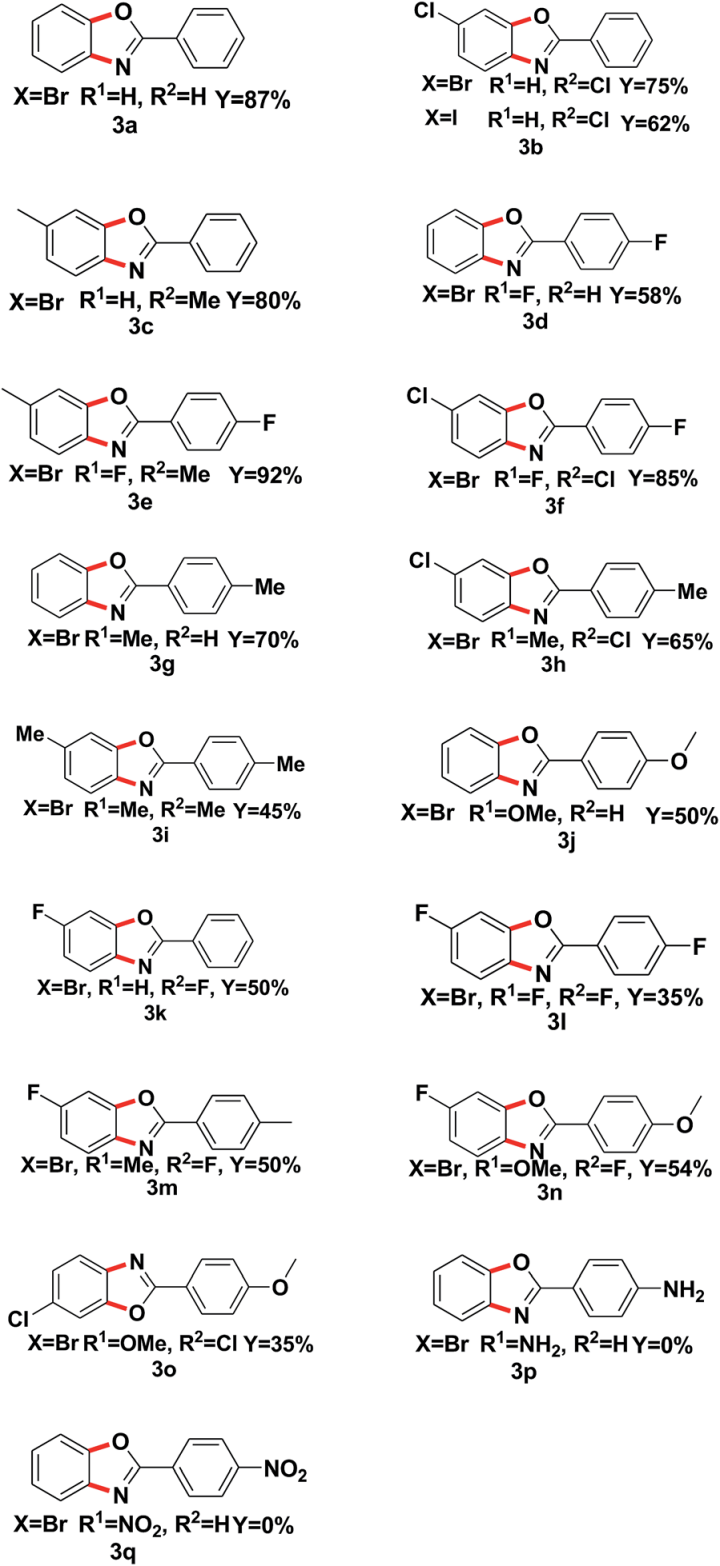

a Reaction conditions: 1a (0.5 mmol), *o*-dihalo substrate (1.5 eq.), Fe_2_O_3_ (20% mol), K_2_CO_3_ (1 eq.), DMEDA (20%) were added to PhMe (2 mL) and react at 110 °C for 48 h under N_2_.

Based on the results observed in the current study and Goldberg reaction,^15^ we have proposed a reaction mechanism for this transformation, which is shown in Scheme 3. The initial transmetalation of benzamide with Fe_2_O_3_L_*n*_ in the presence of K_2_CO_3_ would give rise to the iron(iii) species A. Complex A would then undergo an oxidative addition reaction with 1-bromo-2-iodobenzene to give the iron(v) species B, which would undergo a reductive elimination reaction to give iron(iii) species C with the concomitant formation of a C–N bond. Followed the tautomerism of intermediate C to D, the intermediate iron(iii) species E was formed in the presence of K_2_CO_3_, which would undergo another oxidative addition reaction to afford iron(v) species F. Compound 3a would then be obtained *via* a reductive elimination reaction from iron(v) species F.

**Scheme 3:**
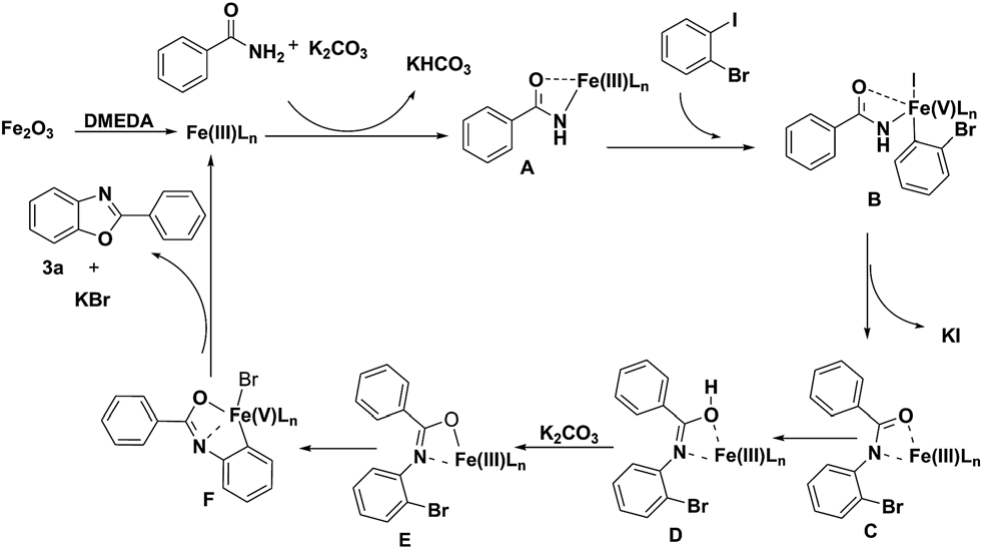
Possible catalytic cycle.

In summary, we have demonstrated that the cheap and environmental friendly catalyst system composed of Fe_2_O_3_ and ligand DMEDA is highly effective for the synthesis of 2-arylbenzoxazoles. The new catalyzed system can be effective for both C–N coupling and C–O coupling.

## Conflicts of interest

There are no conflicts to declare.

## Supplementary Material

RA-008-C7RA13080E-s001
