# Supplementary material for: Synthesis of benzoxazoles via an iron-catalyzed domino C–N/C–O cross-coupling reaction
Source: RSC Adv. 2018 Jan 9;8(5):2267–70. doi: 10.1039/c7ra13080e (PMC9077259; doi:10.1039/c7ra13080e)
Supplement: RA-008-C7RA13080E-s001 [file RA-008-C7RA13080E-s001.pdf]

Electronic Supplementary Material (ESI) for RSC Advances.
This journal is © The Royal Society of Chemistry 2018

Synthesis of Benzoxazoles via Iron-Catalyzed Domino C–N/C–O Cross-Coupling Reaction

Bo Yang, Weiye Hu and Songlin Zhang*

Key Laboratory of Organic Synthesis of Jiangsu Province, College of Chemistry, Chemical Engineering and Materials Science, Soochow University, Suzhou 215123, P. R. China

Table of contents:

General Information S2

Synthesis of 2-phenylbenzo[*d*]oxazole. General procedure S2

^{1}H and ^{13}C of all compounds S6

General Information

All of the reagents and solvents were used directly as obtained commercially unless otherwise noted.Petroleum ether (PE) used refers to the 60-90 ^{0}C boiling point fraction of petroleum. Column chromatography was performed with 300-400 mesh silica gel using flash column techniques. ^{1}H and ^{13}C NMR spectra were determined in $CDCl_3$ on a Varian-Inova 400MHz spectrometer and chemical shifts were measured relative to the signals for residual chloroform (7.26 ppm) in the deuterated solvent, unless otherwise stated. Chemical shifts in ^{13}C NMR spectra are reported relative to the central line of the DMSO (δ = 77.00 ppm).

Synthesis of 2-phenylbenzo[*d*]oxazole. General procedure.

Benzamide (0.5 mmol), Fe_2O_3 (0.1 mmol), and K_2CO_3 (0.5 mmol) were weighed into an oven-dried Schlenk tube which was sealed with a plug and an nitrogen atmosphere was established. Then, DMEDA (0.1 mmol), 1,2-dibromobenzene (0.45 mmol) and PhMe (2 mL) was added via syringe. The Schlenk tube was heated to 110 ºC and stirring for 48 h. When the reaction complete, the heterogeneous mixture was cooled to room temperature and diluted with water and ethoxyethane. The organic solution was washed with brine, dried (Na_2SO_4), and purified by column chromatography on silica gel (eluting with 20:1 petroleum ether:ethyl acetate) to give desired 2-phenylbenzo[*d*]oxazole.

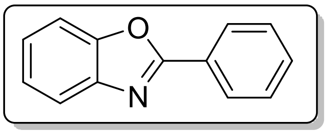

2-phenylbenzo[*d*]oxazole(3a): white solid; yield: 84.8 mg (87%); mp=102-104℃; ^{1}H NMR (400 MHz, $CDCl_3$): δ=8.26-8.24 (m, 2H), 7.78-7.76 (m, 1H), 7.58-7.55 (m, 1H), 7.52-7.49 (m, 3H), 7.36-7.33 (m, 2H); ^{13}C NMR (100 MHz, $CDCl_3$): δ =163.0, 150.7, 142.1, 131.5, 128.9, 127.6, 127.1, 125.1, 124.5, 120.0, 110.5; HRMS(EI) calcd for $C_{13}H_9NO$ (M^+): 195.0684; found: 195.0686.

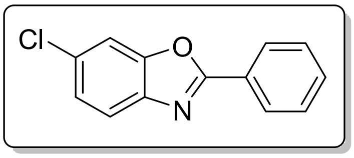

6-chloro-2-phenylbenzo[*d*]oxazole(3b)

white solid; mp=107-108℃; ^{1}H NMR (400 MHz, $CDCl_3$): δ=8.23-8.21 (m, 2H), 7.67 (d, J=8.48 Hz, 1H), 7.59-7.50 (m, 4H), 7.34-7.32 (m, 1H); ^{13}C NMR (100 MHz, $CDCl_3$): δ=163.7, 150.9, 140.9, 131.8, 130.7, 129.0, 127.6, 126.7, 125.3, 120.5, 111.2; HRMS(EI) calcd for $C_{13}H_8{}^{35}ClNO$ (M^+): 229.0294; found: 229.0294.

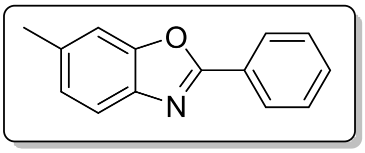

6-methyl-2-phenylbenzo[*d*]oxazole(3c)

white solid; yield: 83.6 mg (80%); mp=94-94.5℃; ^{1}H NMR (400 MHz, $CDCl_3$): δ =8.24-8.22 (m,

2H), 7.64 (d, J=8.08 Hz, 1H), 7.51 (s, 3H), 7.37 (s, 1H), 7.16 (d, J=7.96 Hz, 1H), 2.50 (s, 3H); ^{13}C NMR (100 MHz, $CDCl_3$): δ=162.5, 151.0, 139.9, 135.5, 131.2, 128.8, 127.4, 127.3, 125.8, 119.3, 110.7, 21.8; HRMS(EI) calcd for $C_{14}H_{11}NO$ (M^+): 209.0841; found: 209.0838.

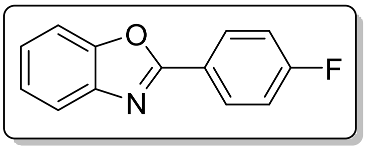

2-(4-fluorophenyl)benzo[*d*]oxazole(3d)

white solid; yield: 61.8 mg (58%). mp=92-95℃; ^{1}H NMR (400 MHz, $CDCl_3$): δ= 8.27-8.23 (m, 2H), 7.77-7.75 (m, 1H), 7.58-7.56 (m, 1H), 7.36-7.34 (m, 2H), 7.21 (t, J=8.64 Hz, 2H); ^{13}C NMR (100 MHz, $CDCl_3$): δ=164.8 (d, $^1J_{C-F}$=251 Hz), 162.1, 150.8, 142.0, 129.8 (d, $^3J_{C-F}$=8.82 Hz), 159.1, 124.6, 123.5, 120.0, 116.2 (d, $^2J_{C-F}$= 22.06 Hz), 110.6; HRMS(EI) calcd for $C_{13}H_8FNO$ (M^+): 213.0590; found: 213.0585.

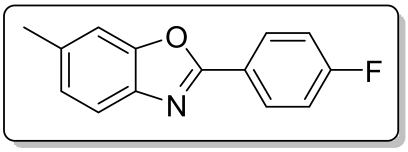

2-(4-fluorophenyl)-6-methylbenzo[*d*]oxazole(3e)

white solid; yield: 104 mg (92%). mp=113-116℃; ^{1}H NMR (400 MHz, $CDCl_3$): δ =8.21 (s, 2H), 7.61 (d, J=7.96Hz, 1H), 7.35 (s, 1H), 7.17 (q, J=8.28 Hz, 3H), 2.49 (s, 3H); ^{13}C NMR (100 MHz, $CDCl_3$): δ=164.6 (d, $^1J_{C-F}$=250.9 Hz), 161.6, 151.0, 139.8, 135.6, 129.6 (d, $^3J_{C-F}$=8.75 Hz), 125.8, 123.7, 119.3, 116.1 (d, $^2J_{C-F}$=22.02 Hz), 110.7, 21.8; HRMS(EI) calcd for $C_{14}H_{11}NO$ (M^+): 227.0746; found: 227.0740.

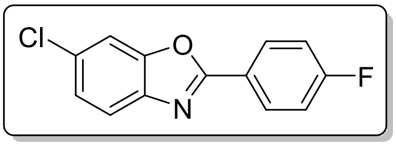

6-chloro-2-(4-fluorophenyl)benzo[*d*]oxazole(3f)

white solid; yield: 105 mg (85%). mp=132-133℃; ^{1}H NMR (400 MHz, $CDCl_3$): δ =8.22 (t, J=5.56 Hz, 2H), 7.65 (d, J=8.4 Hz, 1H), 7.56 (s, 1H), 7.33 (d, J=8.16 Hz, 1H), 7.21 (t, J=8.36 Hz, 2H); ^{13}C NMR (100 MHz, $CDCl_3$): δ=165.0 (d, $^1J_{C-F}$=251.84 Hz), 162.8, 150.9, 140.8, 130.7, 129.9 (d, $^3J_{C-F}$=8.92 Hz), 125.4, 123.0, 120.4, 116.3 (d, $^2J_{C-F}$=22.08 Hz), 111.2; HRMS(EI) calcd for $C_{14}H_{11}NO$ (M^+): 247.0200; found: 247.0202.

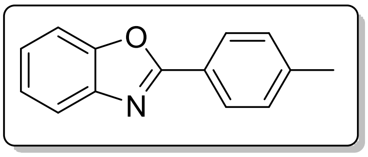

2-p-tolylbenzo[*d*]oxazole(3g)

white solid; yield: 73.2 mg (70%). mp=116-117℃; ^{1}H NMR (400 MHz, $CDCl_3$): δ =8.13 (d, J=8.2 Hz, 2H), 7.76-7.74 (m, 1H), 7.56-7.54 (m, 1H), 7.35-7.30 (m, 4H), 2.42 (s, 3H); ^{13}C NMR (100 MHz, $CDCl_3$): δ=163.2, 150.6, 142.1, 142.0, 129.6, 127.5, 124.8, 124.4, 124.3, 119.8, 110.4, 21.6; HRMS(EI) calcd for $C_{14}H_{11}NO$ (M^+):209.0841; found: 209.0840.

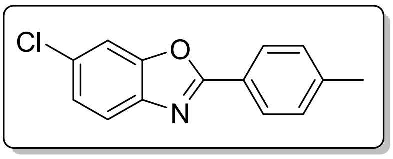

6-chloro-2-p-tolylbenzo[*d*]oxazole(3h)

white solid; yield: 78.9 mg (65%). mp=126-128℃; ^{1}H NMR (400 MHz, $CDCl_3$): δ =8.09 (d, J=8.24 Hz, 2H), 7.64 (d, J= 8.48 Hz, 1H), 7.55 (s, 1H), 7.32-7.29 (m, 3H), 2.43 (s, 3H); ^{13}C NMR (100 MHz, $CDCl_3$): δ=163.9, 150.8, 142.4, 140.9, 130.4, 129.7, 127.6, 125.1, 123.9, 120.2, 111.1, 21.6; HRMS(EI) calcd for $C_{14}H_{10}ClNO$ (M^+): 243.0451; found: 243.0450.

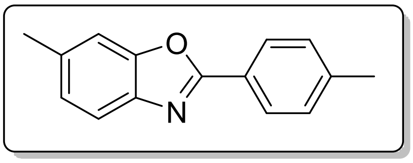

6-methyl-2-p-tolylbenzo[*d*]oxazole(3i)

white solid; yield: 50.18 mg (45%). mp=103-104℃; ^{1}H NMR (400 MHz, $CDCl_3$): δ=8.11 (d, J=8.2 Hz, 2H), 7.62 (d, J=8.08 Hz, 1H), 7.36 (s, 1H), 7.31 (d, J=8.0 Hz, 2H), 7.15 (d, J=8.08 Hz, 1H), 2.49 (s, 3H), 2.42 (s, 3H); ^{13}C NMR (100 MHz, $CDCl_3$): δ=162.8, 151.0, 141.7, 140.0, 135.3, 129.6, 127.4, 125.7, 124.6, 119.1, 110.7, 21.8, 21.6; HRMS(EI) calcd for $C_{15}H_{13}NO$ (M^+): 223.0997; found: 223.0991.

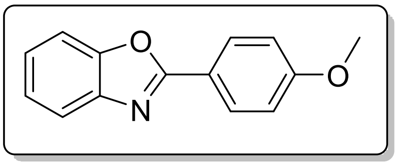

2-(4-methoxyphenyl)benzo[*d*]oxazole(3j)

white solid; yield: 56.2 mg (50%). mp=101℃; ^{1}H NMR (400 MHz, $CDCl_3$): δ=8.19 (d, J= 8.92 Hz, 2H), 7.75-7.72 (m, 1H), 7.56-7.53 (m, 1H), 7.35-7.30 (m, 2H), 7.01 (d, J=8.88 Hz, 2H), 3.87 (s, 3H); ^{13}C NMR (100 MHz, $CDCl_3$): δ=163.1, 162.2, 150.6, 142.2, 129.3, 124.5, 124.4, 119.6, 119.6, 114.3, 110.3, 55.4; HRMS(EI) calcd for $C_{14}H_{11}NO_2$ (M^+): 225.0790; found: 225.0791.

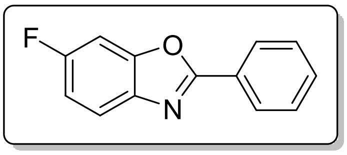

6-fluoro-2-phenylbenzo[*d*]oxazole(3k)

white solid; yield: 53.2 mg (50%). mp=109-110℃; ^{1}H NMR (400 MHz, $CDCl_3$): δ=8.31-8.28 (m, 2H), 7.77 (q, J=4.88 Hz, 1H), 7.61-7.59 (m, 3H), 7.40-7.37 (m, 1H), 7.21-7.15 (m, 1H), ^{13}C NMR (100 MHz, $CDCl_3$): δ=163.7, 160.7 (d, $^{1}J_{C-F}$=242.62 Hz), 150.7 (d, $^{3}J_{C-F}$=14.57 Hz), 138.4, 131.6, 129.0, 127.5, 126.9, 120.2 (d, $^{3}J_{C-F}$=10.05), 112.5 (d, $^{2}J_{C-F}$=24.49 Hz), 98.7 (d, $^{2}J_{C-F}$=28.03 Hz); HRMS(EI) calcd for $C_{13}H_8FNO$ (M^+): 213.0590; found: 213.0589.

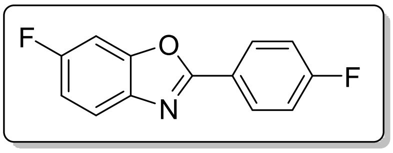

6-fluoro-2-(4-fluorophenyl)benzo[*d*]oxazole(3l)

white solid; yield: 40.4 mg (35%). mp=121-123℃; ^{1}H NMR (400 MHz, $CDCl_3$): δ=8.22-8.18 (m, 2H), 7.69-7.66 (m, 1H), 7.30-7.26 (m, 1H), 7.23-7.18 (m, 2H), 7.13-7.07 (m, 1H); ^{13}C NMR (100 MHz, $CDCl_3$): δ=164.8 (d, $^{1}J_{C-F}$=251.48 Hz), 162.8, 160.6 (d, $^{1}J_{C-F}$=242.81 Hz), 150.7 (d, $^{3}J_{C-F}$=14.67 Hz), 138.3, 129.7 (d, $^{3}J_{C-F}$=8.86 Hz), 123.2 (d, $^{3}J_{C-F}$=3.17 Hz), 120.2 (d, $^{3}J_{C-F}$=9.99 Hz), 116.2 (d,$^{2}J_{C-F}$=22.11 Hz), 112.6 (d, $^{2}J_{C-F}$=24.51 Hz), 98.7 (d, $^{2}J_{C-F}$=28.04 Hz); HRMS(EI) calcd for $C_{13}H_7F_2NO$ (M^+): 231.0496; found: 231.0496.

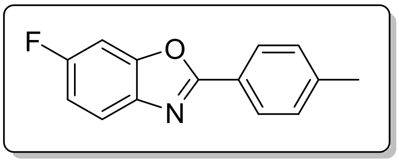

6-fluoro-2-p-tolylbenzo[*d*]oxazole(3m)

white solid; yield: 56.8 mg (50%). mp=125-125.6℃; ^{1}H NMR (400 MHz, $CDCl_3$): δ=8.09 (d, J=8.2 Hz, 2H), 7.67-7.64 (m, 1H), 7.36-7.26 (m, 3H), 7.11-7.05 (m, 1H), 2.42 (s, 3H); ^{13}C NMR (100 MHz, $CDCl_3$): δ=163.9, 160.5 (d, $^{1}J_{C-F}$=242.19 Hz), 150.6 (d, $^{3}J_{C-F}$=14.58 Hz), 142.1, 138.4, 129.7, 127.4, 124.1, 120.0 (d, $^{3}J_{C-F}$=9.98 Hz), 112.3 (d,$^{2}J_{C-F}$=24.47 Hz), 98.6 (d, $^{2}J_{C-F}$=27.99 Hz), 21.6; HRMS(EI) calcd for $C_{14}H_{10}FNO$ (M^+): 227.0746; found: 227.0747.

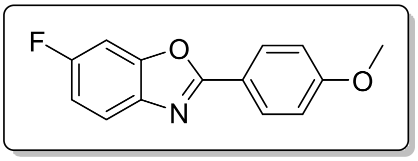

6-fluoro-2-(4-methoxyphenyl)benzo[*d*]oxazole(3n)

white solid; yield: 65.6 mg (54%). mp=155℃; ^{1}H NMR (400 MHz, $CDCl_3$): δ= 8.14-8.11 (m, 2H), 7.65-7.61 (m, 1H), 7.27-7.24 (m, 1H), 7.09-6.98 (m, 3H), 3.87(s, 3H); ^{13}C NMR (100 MHz, $CDCl_3$): δ= 163.7, 162.3, 160.3 (d, $^{1}J_{C-F}$=241.83 Hz), 150.5 (d, $^{3}J_{C-F}$=14.62 Hz), 138.5, 129.2, 119.7 (d, $^{3}J_{C-F}$=10.02 Hz), 114.3, 112.2 (d, $^{2}J_{C-F}$=24.39 Hz), 98.5 (d, $^{2}J_{C-F}$=28.04 Hz), 55.4; HRMS(EI) calcd for $C_{14}H_{10}FNO_2$ (M^+): 243.0696; found: 243.0696.

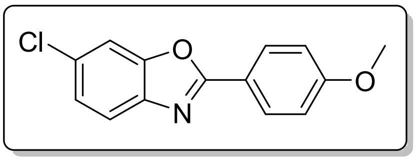

6-chloro-2-(4-methoxyphenyl)benzo[*d*]oxazole(3o)

white solid; yield: 45.3 mg (35%). mp=140-142℃; ^{1}H NMR (400 MHz, $CDCl_3$): δ=8.16 (d, J=8.92 Hz, 2H), 7.62 (d, J=8.44 Hz, 1H), 7.55 (s, 1H), 7.32-7.29 (m, 1H), 7.03-7.01 (m, 2H), 3.89 (s, 3H); ^{13}C NMR (100 MHz, $CDCl_3$): δ=163.8, 162.5, 150.8, 141.1, 130.1, 129.4, 125.1, 120.0, 119.2, 114.4, 111.0, 55.5; HRMS(EI) calcd for $C_{14}H_{10}ClNO_2$ (M^+): 259.0400; found: 259.0404.

1H and 13C of all compounds

3a

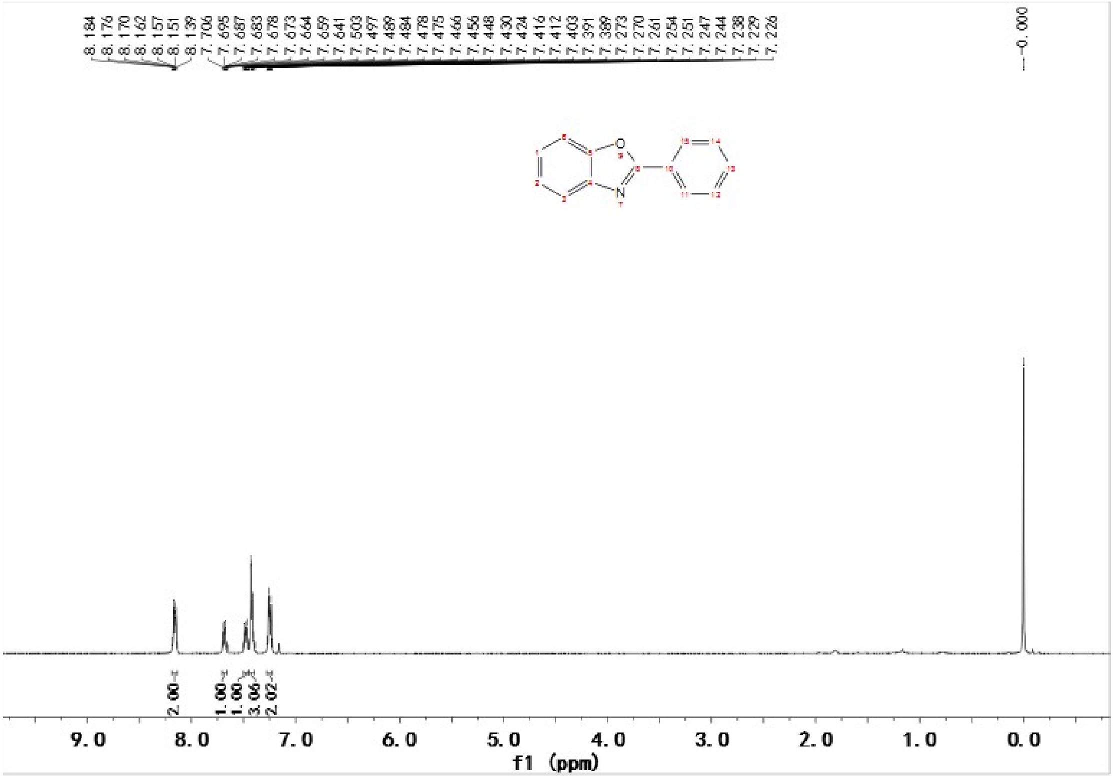

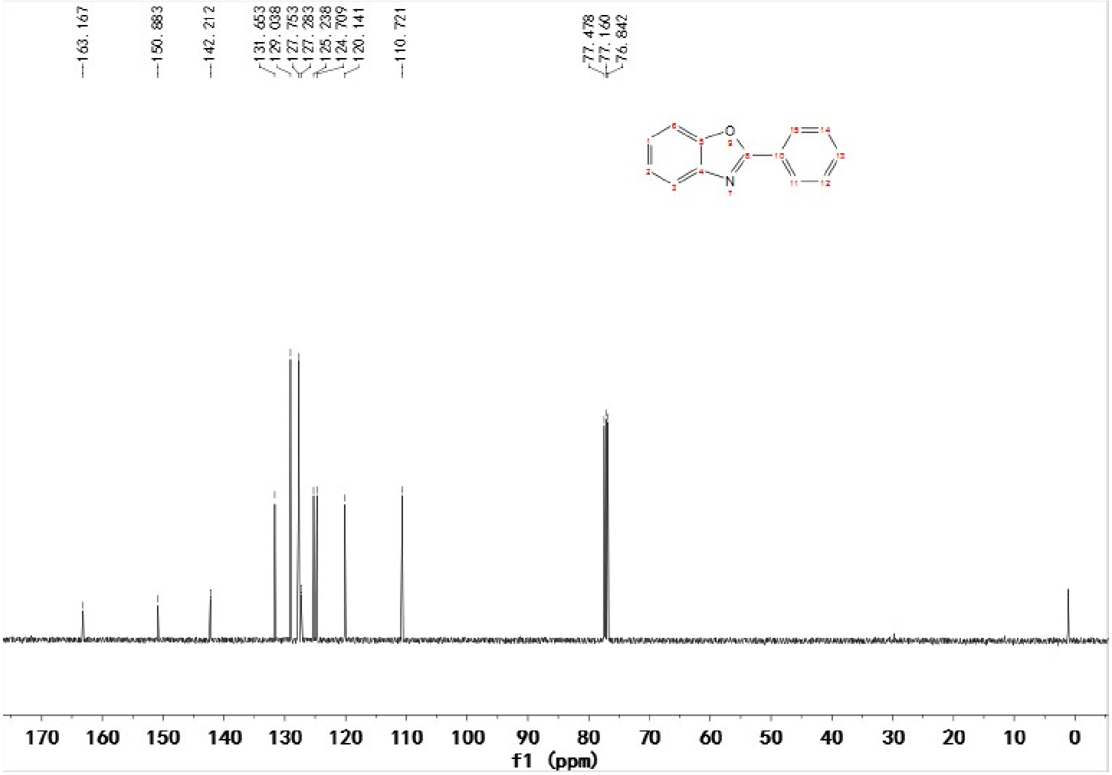

3b

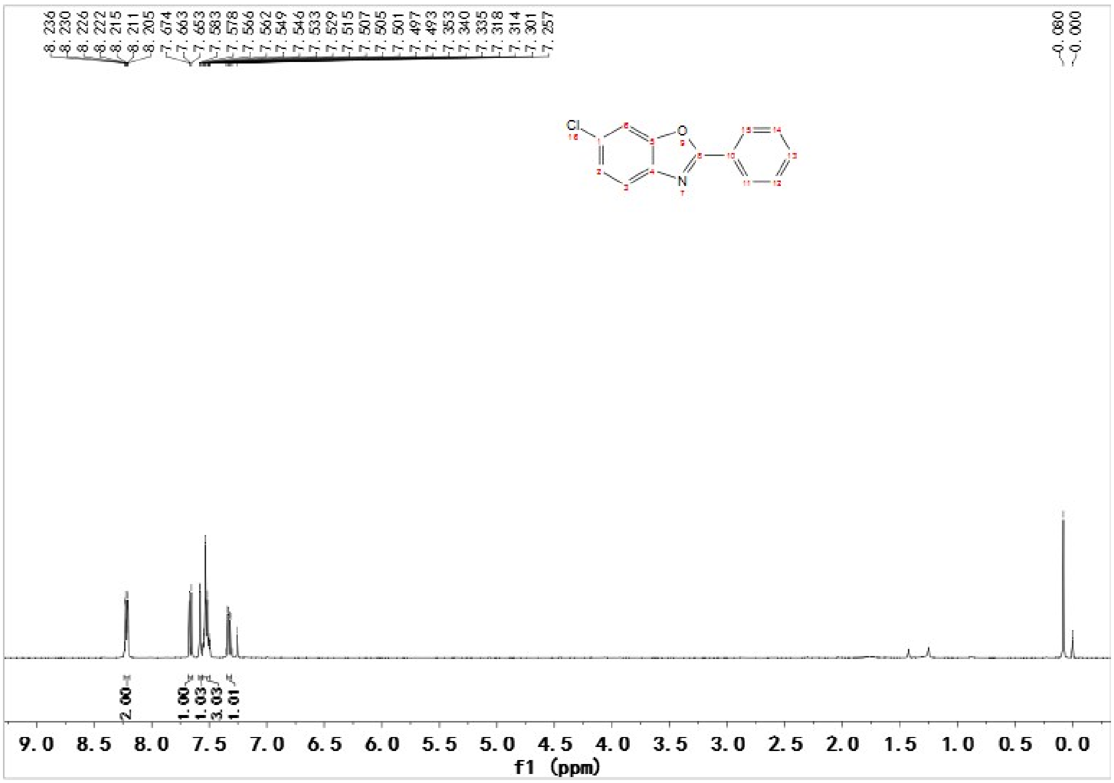

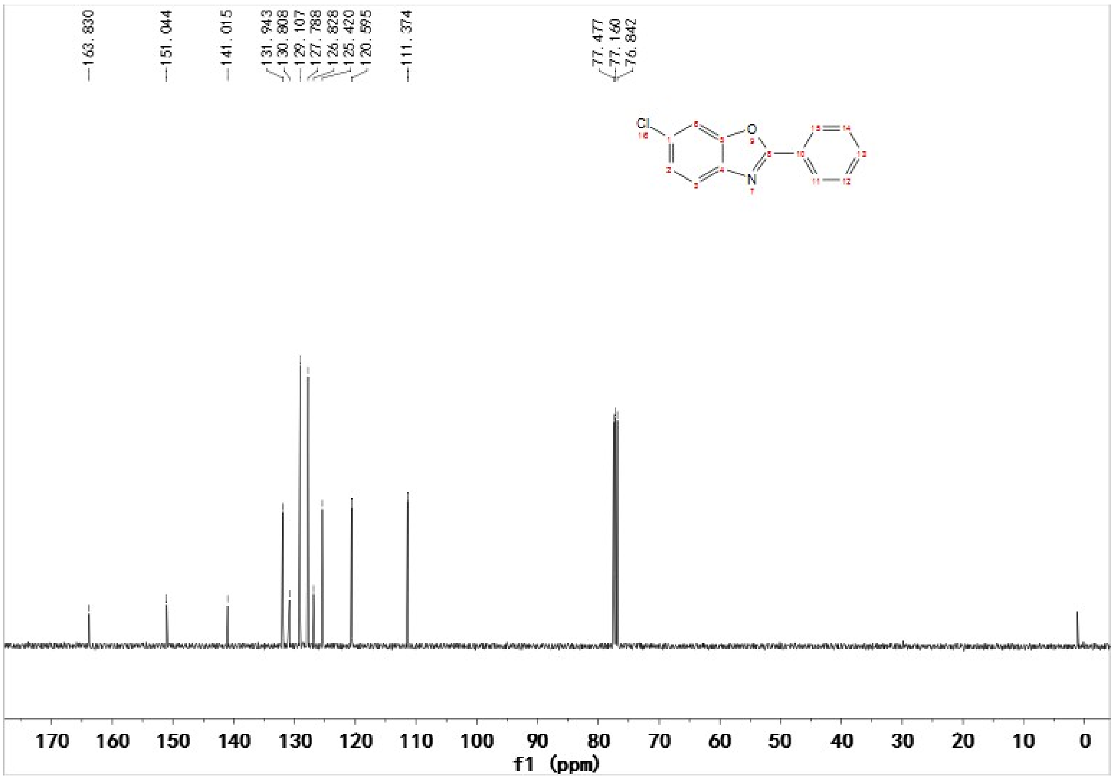

3c

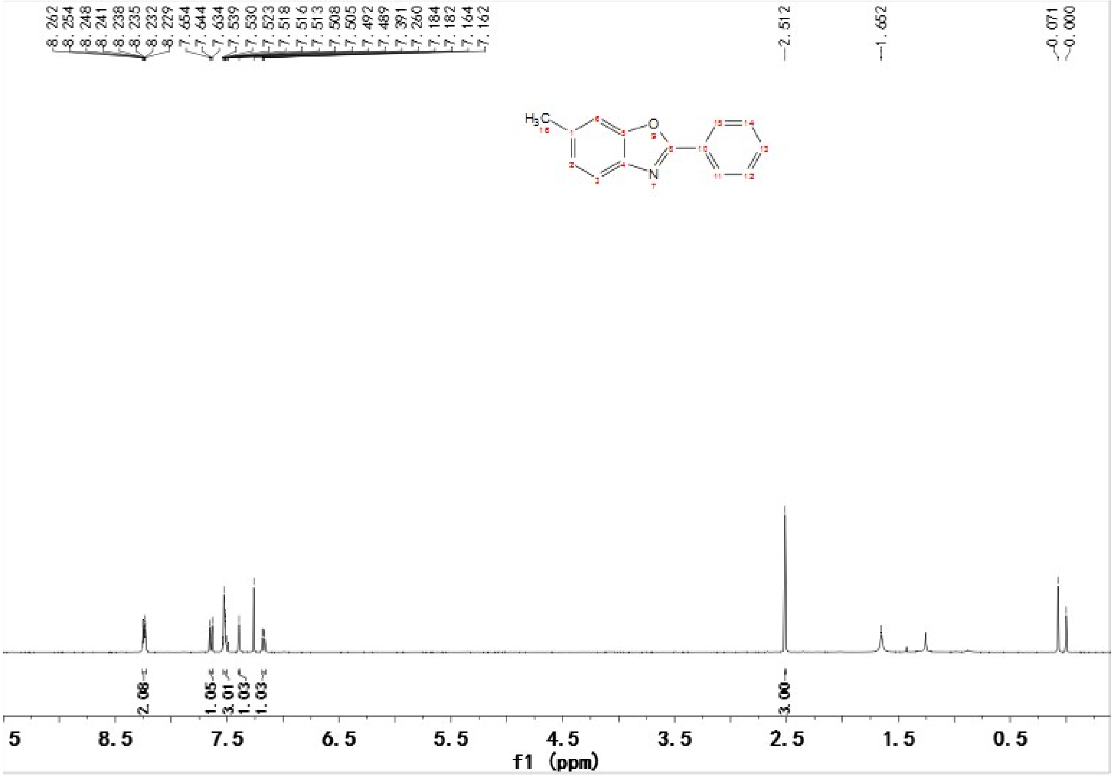
8.262
8.254
8.248
8.241
8.238
8.235
8.232
8.229
7.654
7.644
7.634
7.539
7.530
7.523
7.518
7.516
7.513
7.508
7.505
7.492
7.489
7.391
7.260
7.184
7.182
7.164
7.162
2.512
1.652
0.071
0.000
2.08
1.05
3.01
1.03
1.03
3.00
f1 (ppm)

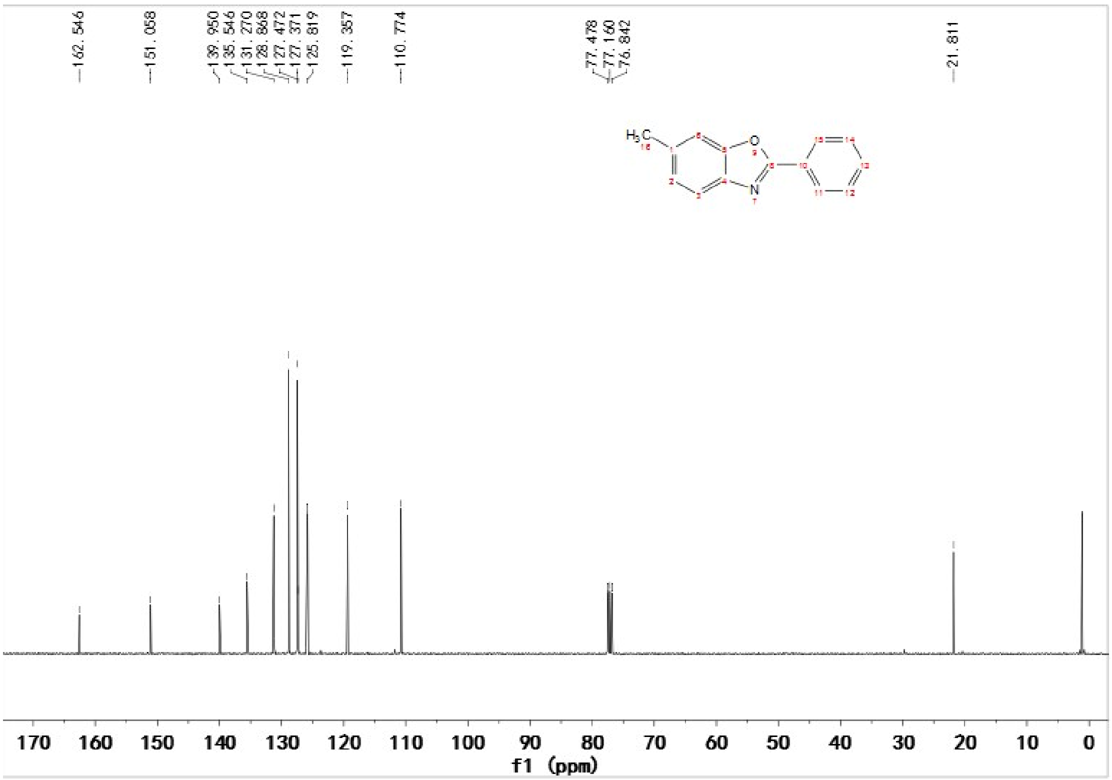
162.546
151.058
139.950
135.546
131.270
128.868
127.472
127.371
125.819
119.357
110.774
77.478
77.160
76.842
21.811
f1 (ppm)

3d

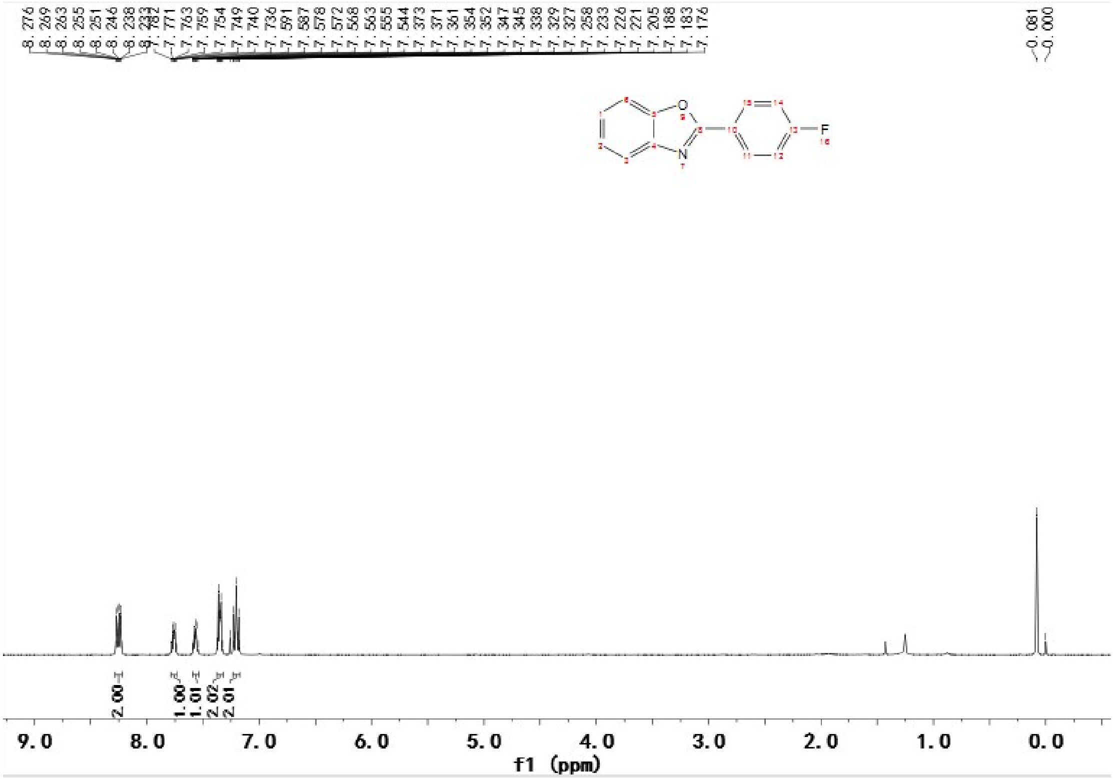

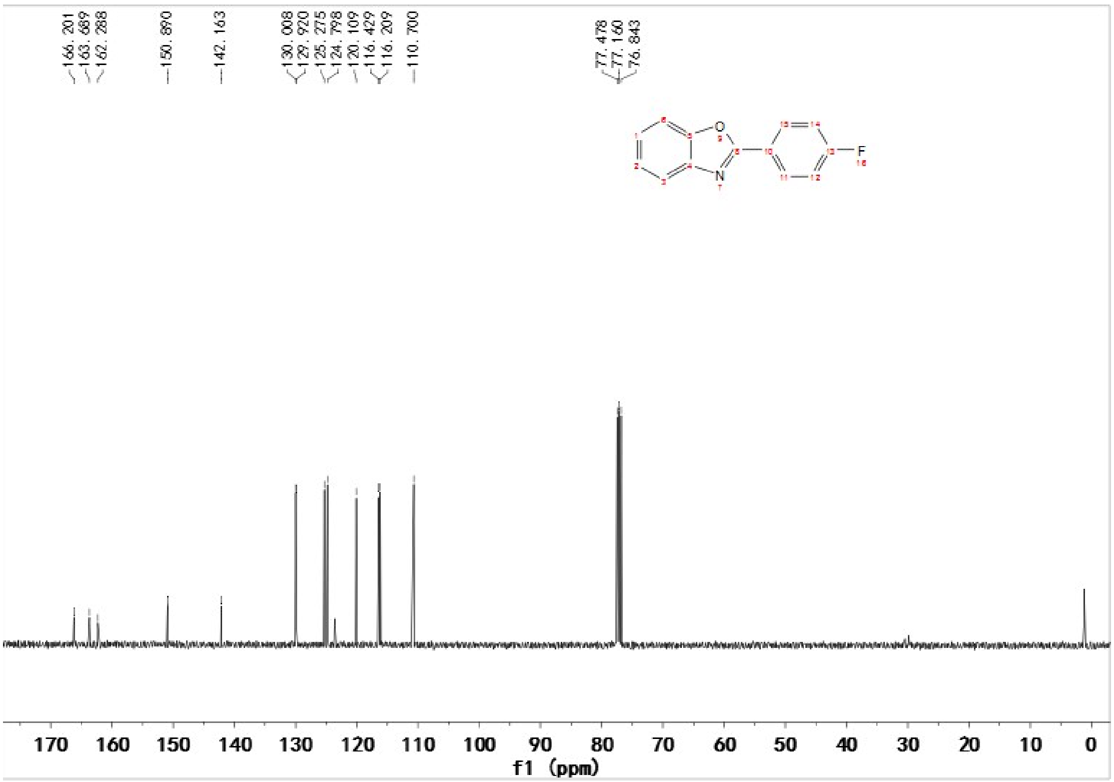

3e

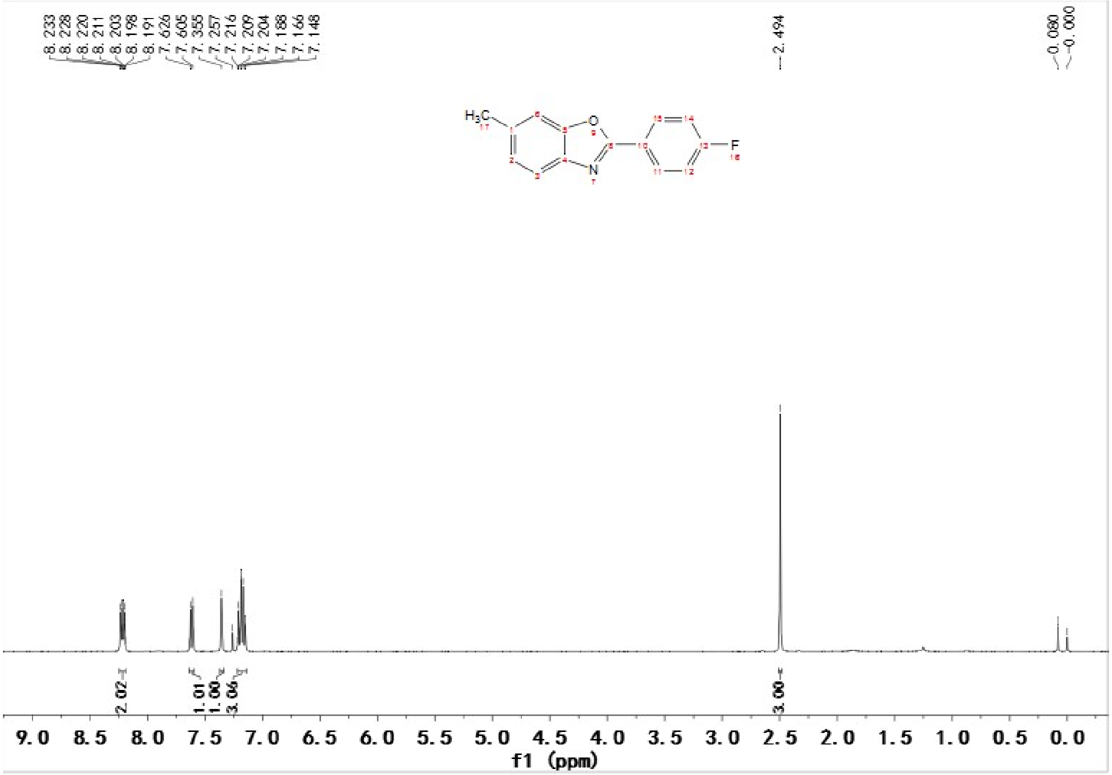
8.233
8.228
8.220
8.211
8.203
8.198
8.191
7.626
7.605
7.355
7.257
7.216
7.209
7.204
7.188
7.166
7.148
2.494
0.080
0.000
H_3C
O
N
F
2.02
1.01
1.00
3.06
3.00
9.0 8.5 8.0 7.5 7.0 6.5 6.0 5.5 5.0 4.5 4.0 3.5 3.0 2.5 2.0 1.5 1.0 0.5 0.0
f1 (ppm)

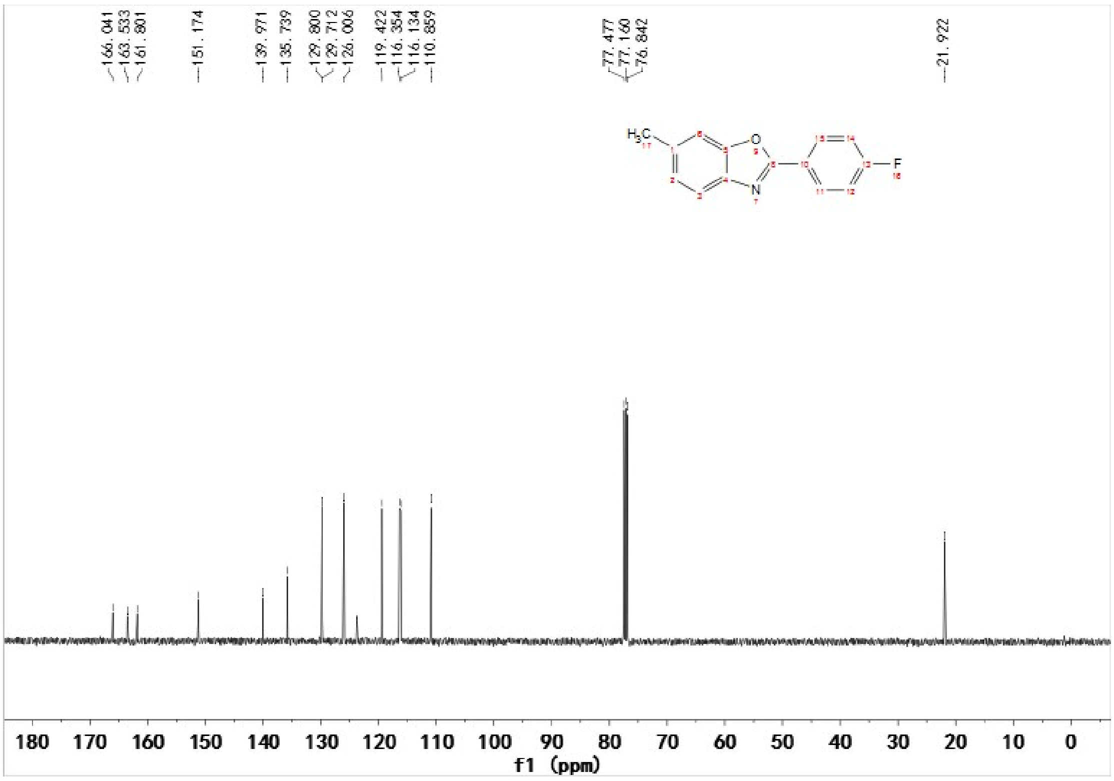
166.041
163.533
161.801
151.174
139.971
135.739
129.800
129.712
126.006
119.422
116.354
116.134
110.859
77.477
77.160
76.842
21.922
H_3C
O
N
F
180 170 160 150 140 130 120 110 100 90 80 70 60 50 40 30 20 10 0
f1 (ppm)

3f

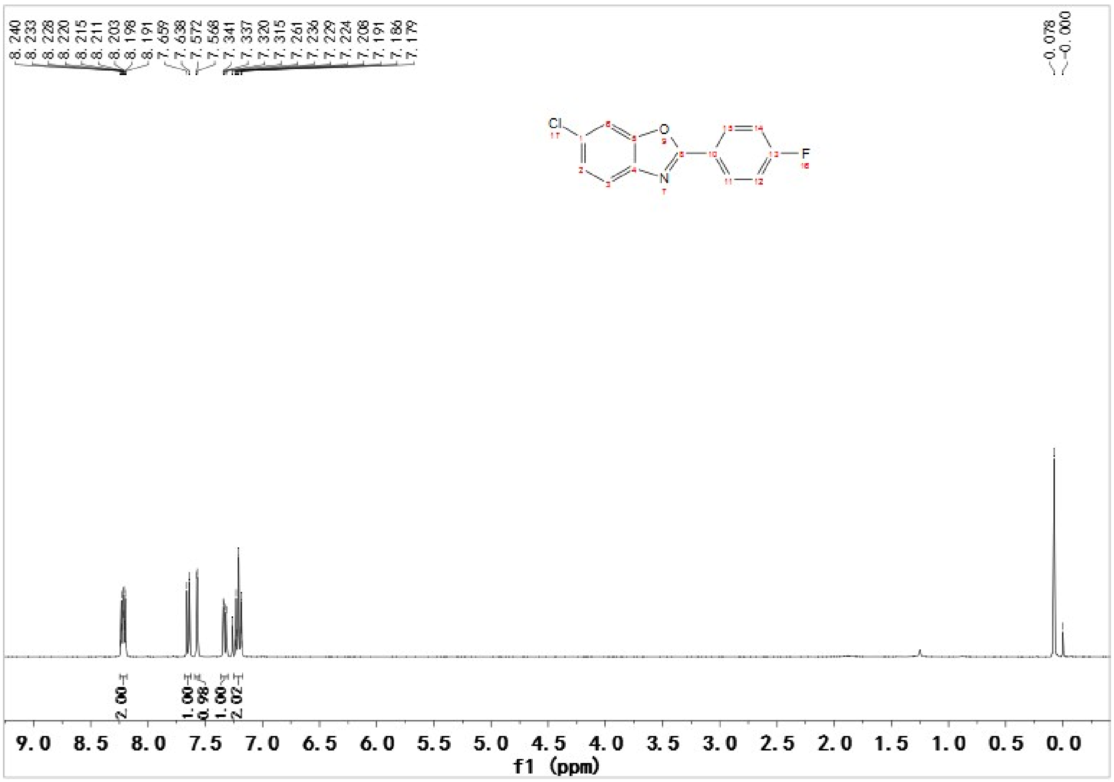
8.240 8.233 8.228 8.220 8.215 8.211 8.203 8.198 8.191 7.659 7.638 7.572 7.568 7.341 7.337 7.320 7.315 7.261 7.236 7.229 7.224 7.208 7.191 7.186 7.179
0.078 -0.000
Cl
O
N
F
2.00
1.00
0.98
1.00
2.02
9.0 8.5 8.0 7.5 7.0 6.5 6.0 5.5 5.0 4.5 4.0 3.5 3.0 2.5 2.0 1.5 1.0 0.5 0.0
f1 (ppm)

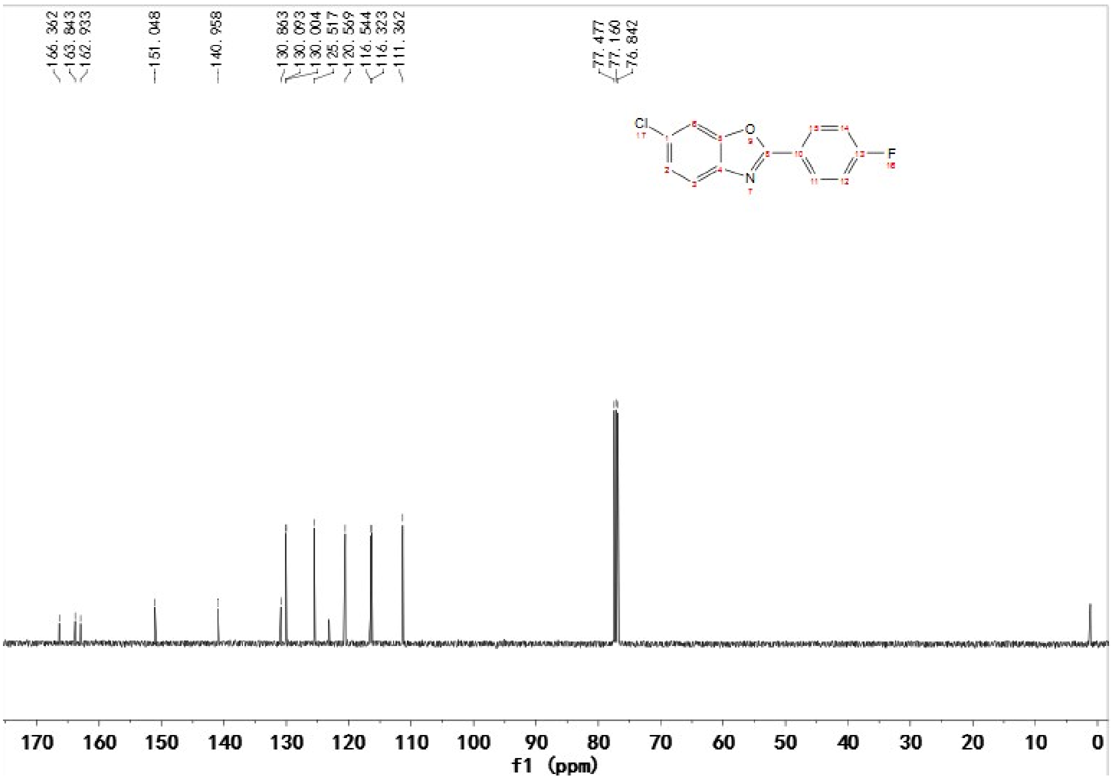
166.362 163.843 162.933
151.048
140.958
130.863 130.093 130.004 125.517 120.569 116.544 116.323 111.362
77.477 77.160 76.842
Cl
O
N
F
170 160 150 140 130 120 110 100 90 80 70 60 50 40 30 20 10 0
f1 (ppm)

3g

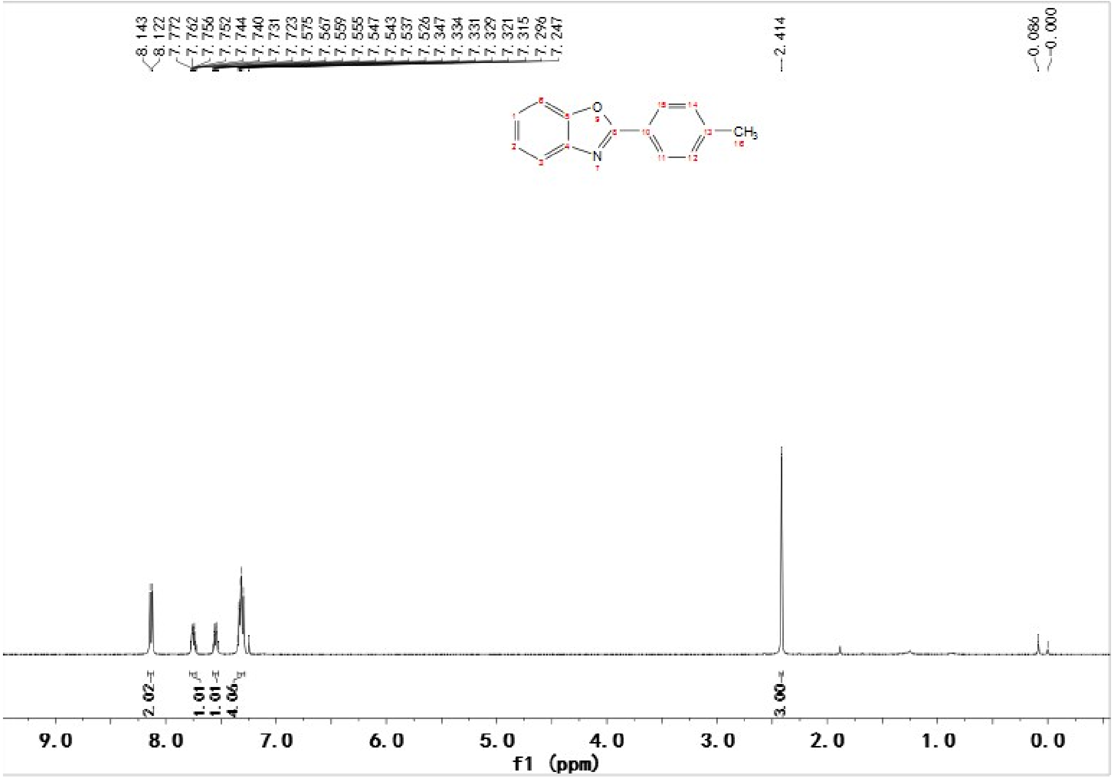

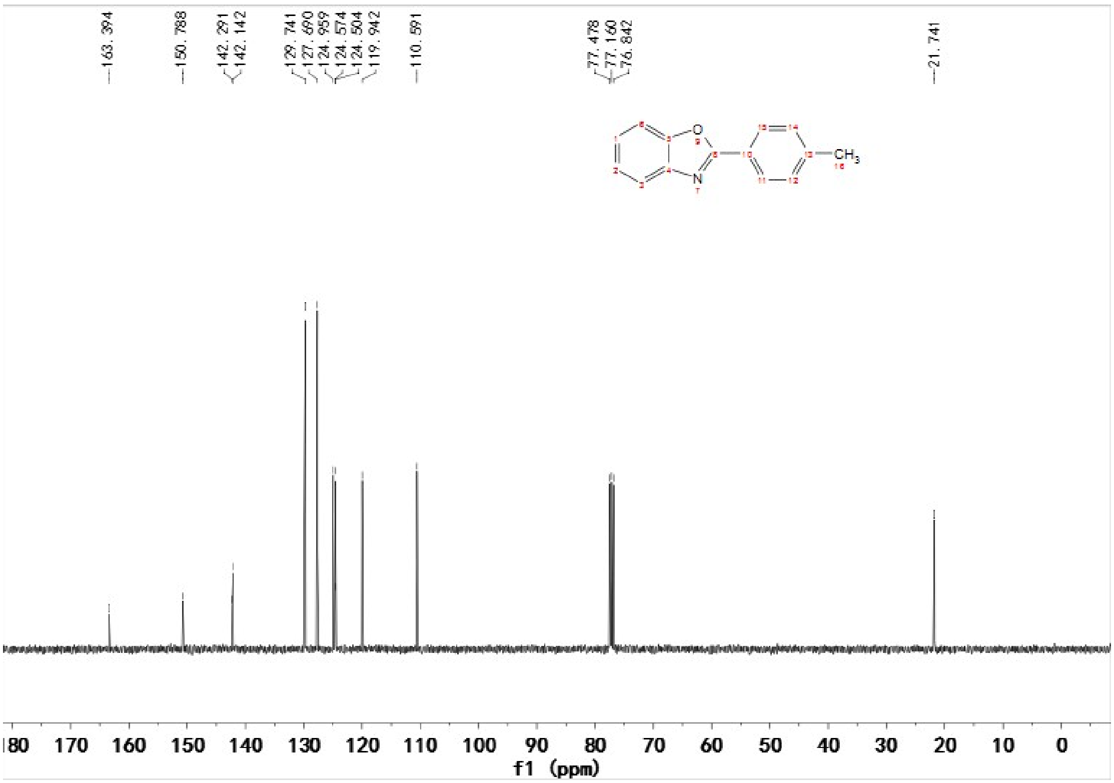

3h

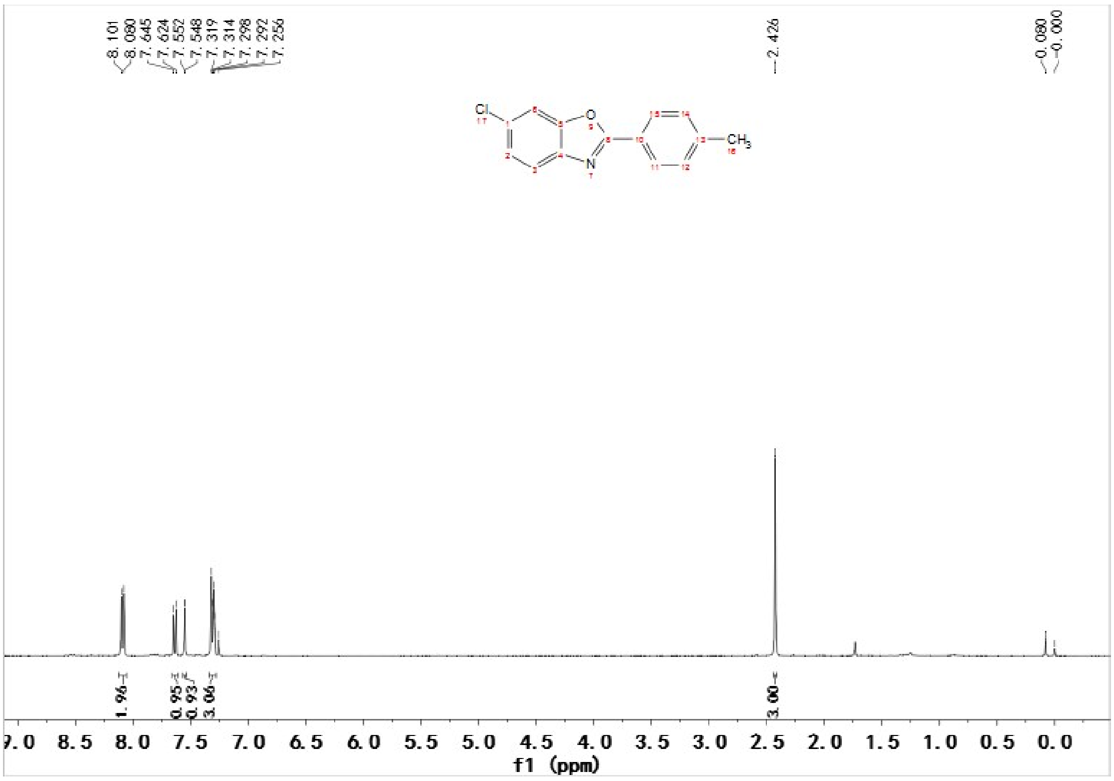

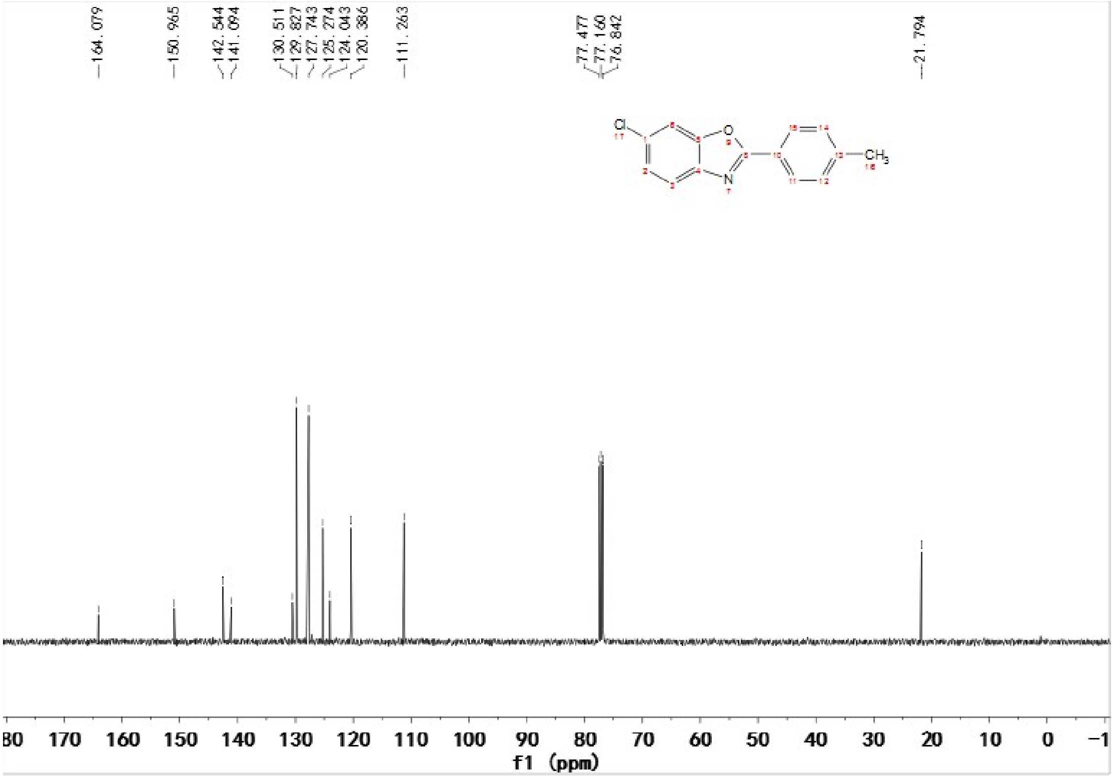

3i

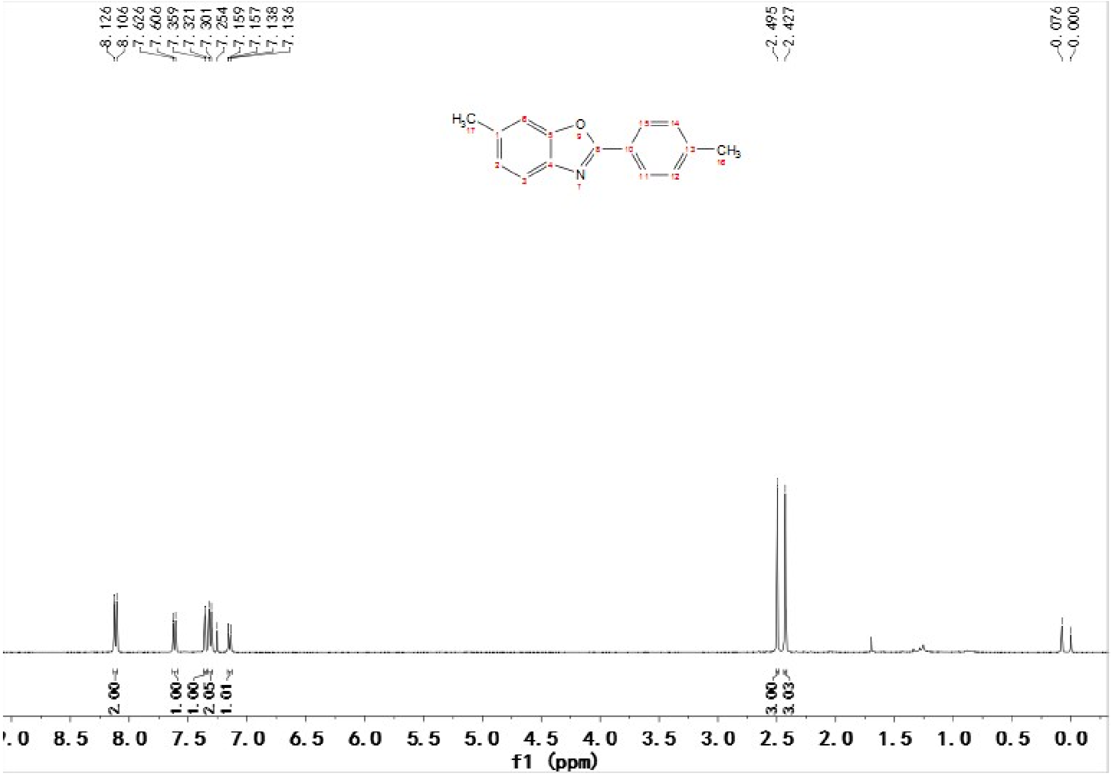
8.126
8.106
7.626
7.606
7.359
7.321
7.301
7.254
7.159
7.157
7.138
7.136
2.495
2.427
0.076
0.000
2.00
1.00
1.00
2.05
1.01
3.00
3.03
f1 (ppm)

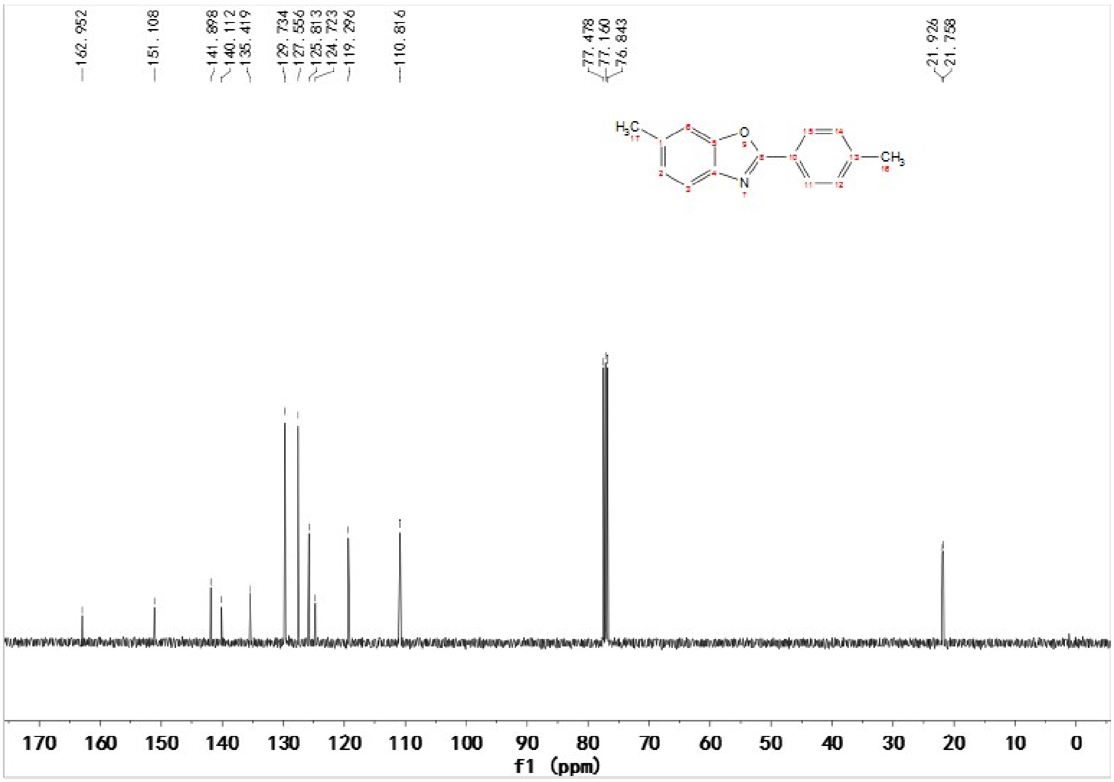
162.952
151.108
141.898
140.112
135.419
129.734
127.556
125.813
124.723
119.296
110.816
77.478
77.160
76.843
21.926
21.758
f1 (ppm)

3j

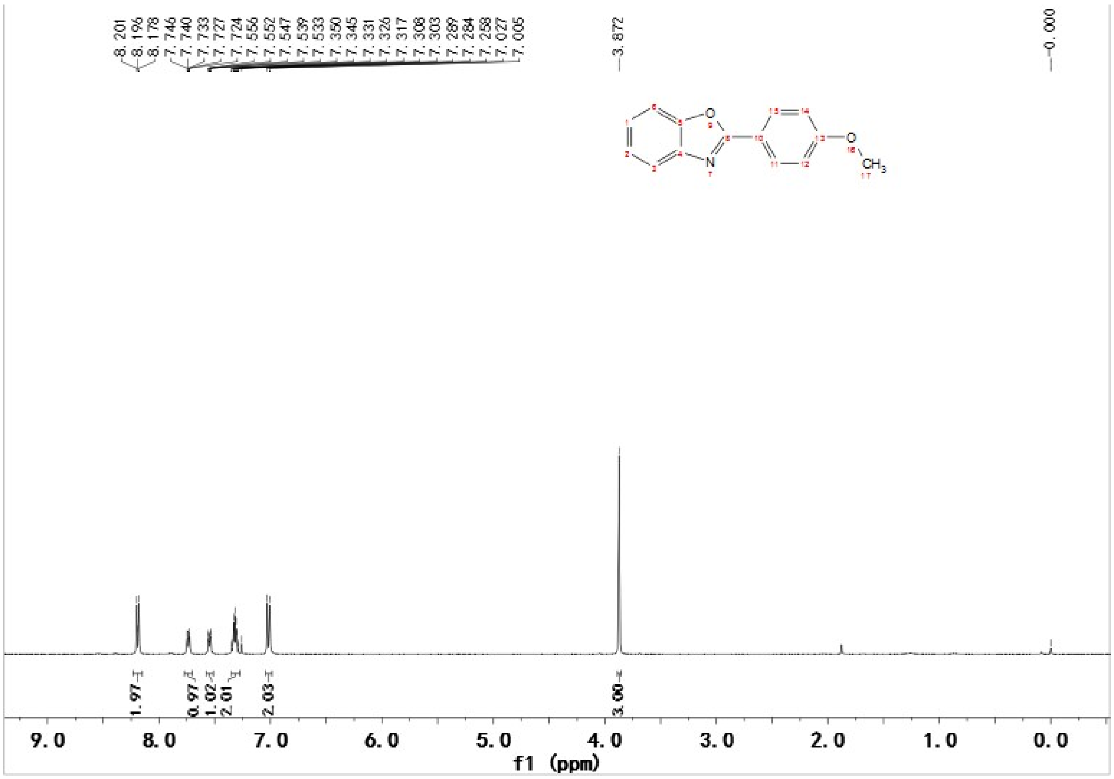
8.201
8.196
8.178
7.746
7.740
7.733
7.727
7.724
7.556
7.552
7.547
7.539
7.533
7.350
7.345
7.331
7.326
7.317
7.308
7.303
7.289
7.284
7.258
7.027
7.005
3.872
0.000
1.97
0.97
1.02
2.01
2.03
3.00
9.0 8.0 7.0 6.0 5.0 4.0 3.0 2.0 1.0 0.0
f1 (ppm)

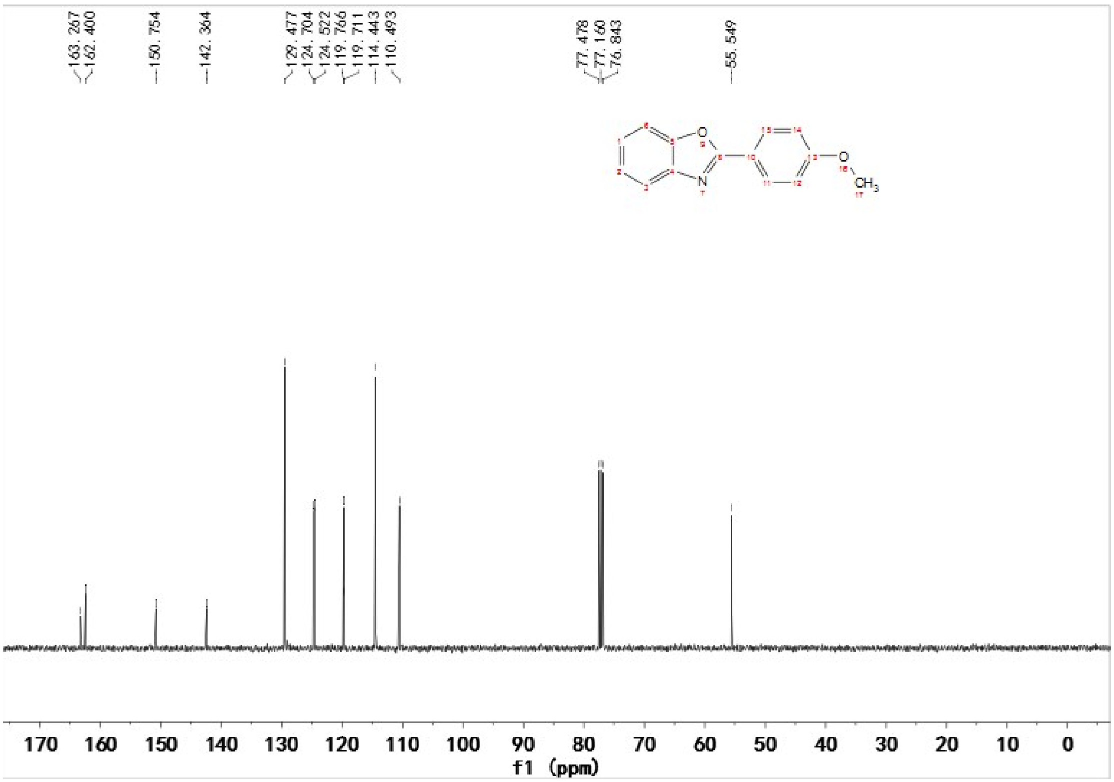
163.267
162.400
150.754
142.364
129.477
124.704
124.522
119.766
119.711
114.443
110.493
77.478
77.160
76.843
55.549
170 160 150 140 130 120 110 100 90 80 70 60 50 40 30 20 10 0
f1 (ppm)

3k

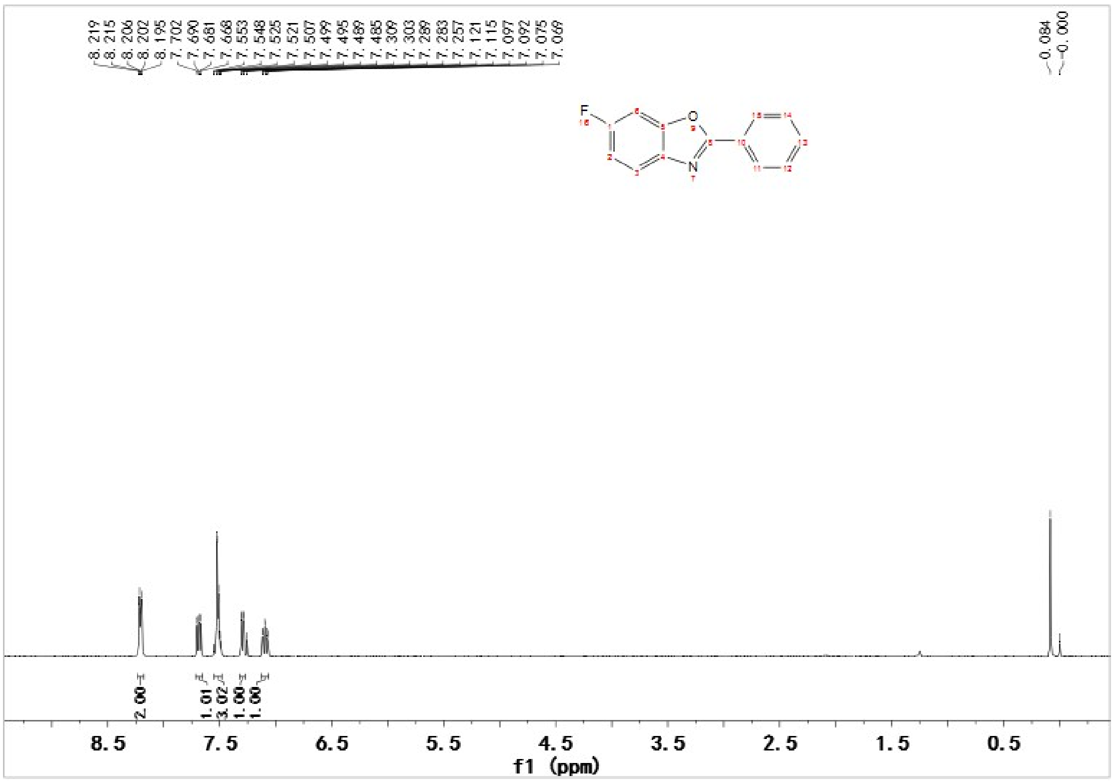
8.219
8.215
8.206
8.202
8.195
7.702
7.690
7.681
7.668
7.553
7.548
7.525
7.521
7.507
7.499
7.495
7.489
7.485
7.309
7.303
7.289
7.283
7.257
7.121
7.115
7.097
7.092
7.075
7.069
0.084
-0.000
F
O
N
2.00
1.01
3.02
1.00
1.00
8.5
7.5
6.5
5.5
4.5
3.5
2.5
1.5
0.5
f1 (ppm)

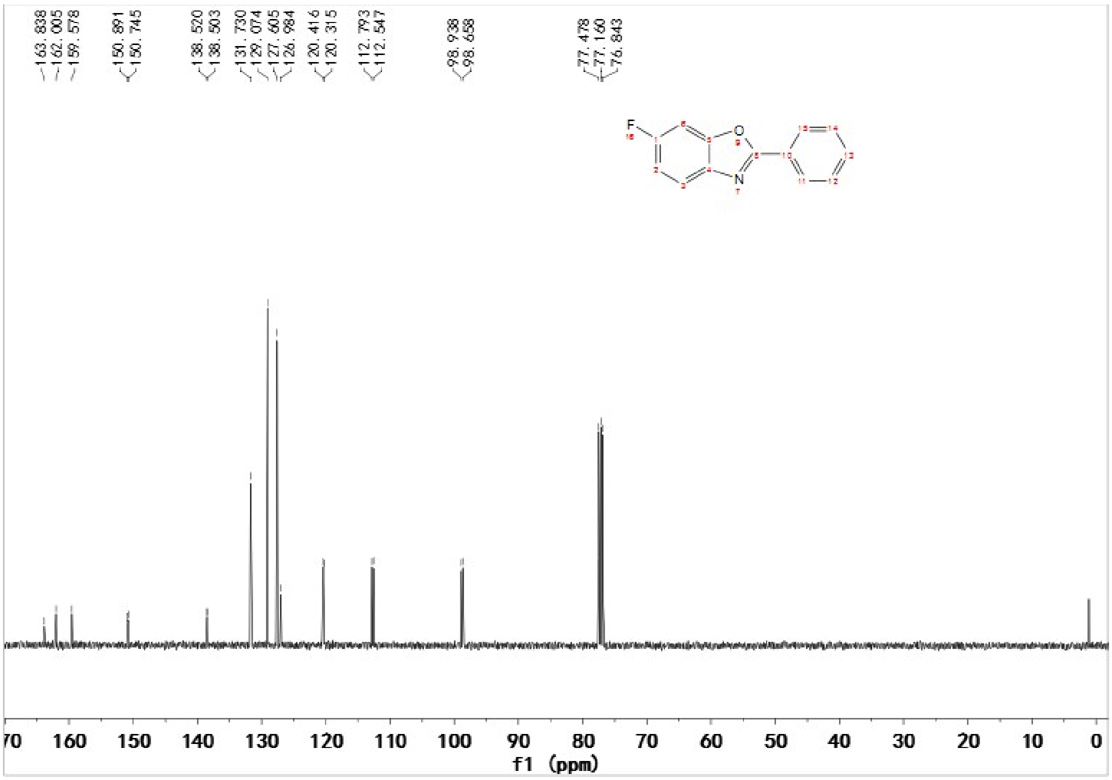
163.838
162.005
159.578
150.891
150.745
138.520
138.503
131.730
129.074
127.605
126.984
120.416
120.315
112.793
112.547
98.938
98.658
77.478
77.160
76.843
F
O
N
70
160
150
140
130
120
110
100
90
80
70
60
50
40
30
20
10
0
f1 (ppm)

3l

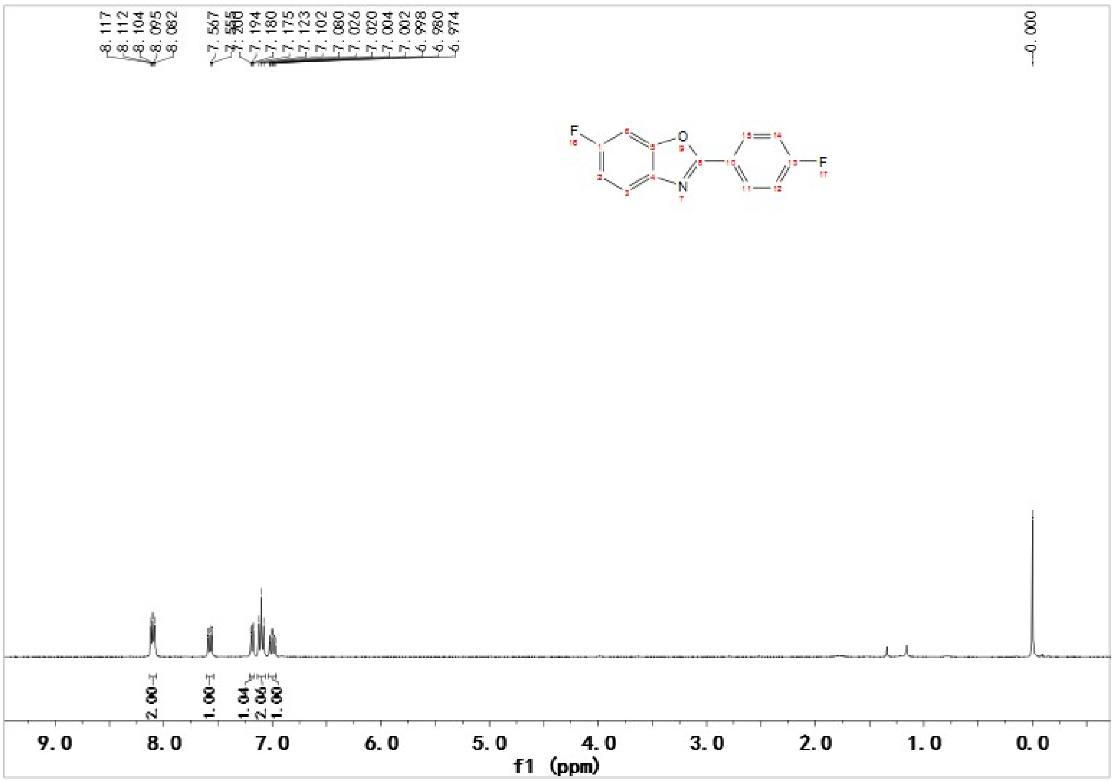

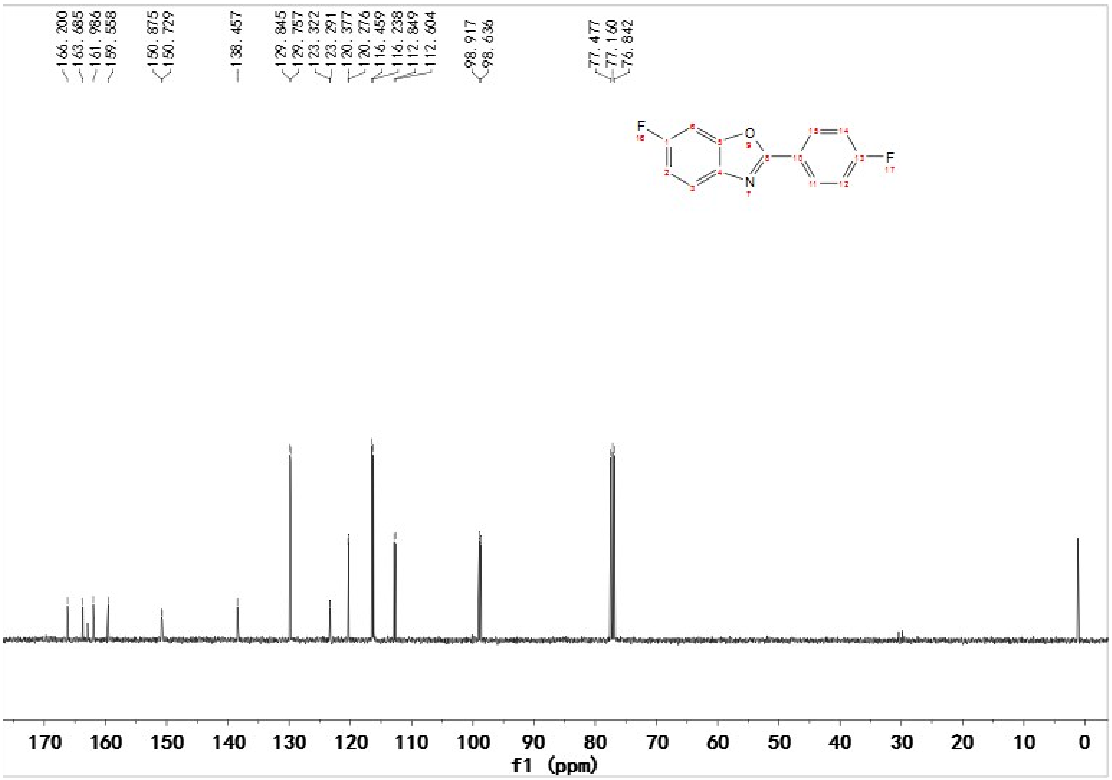

3m

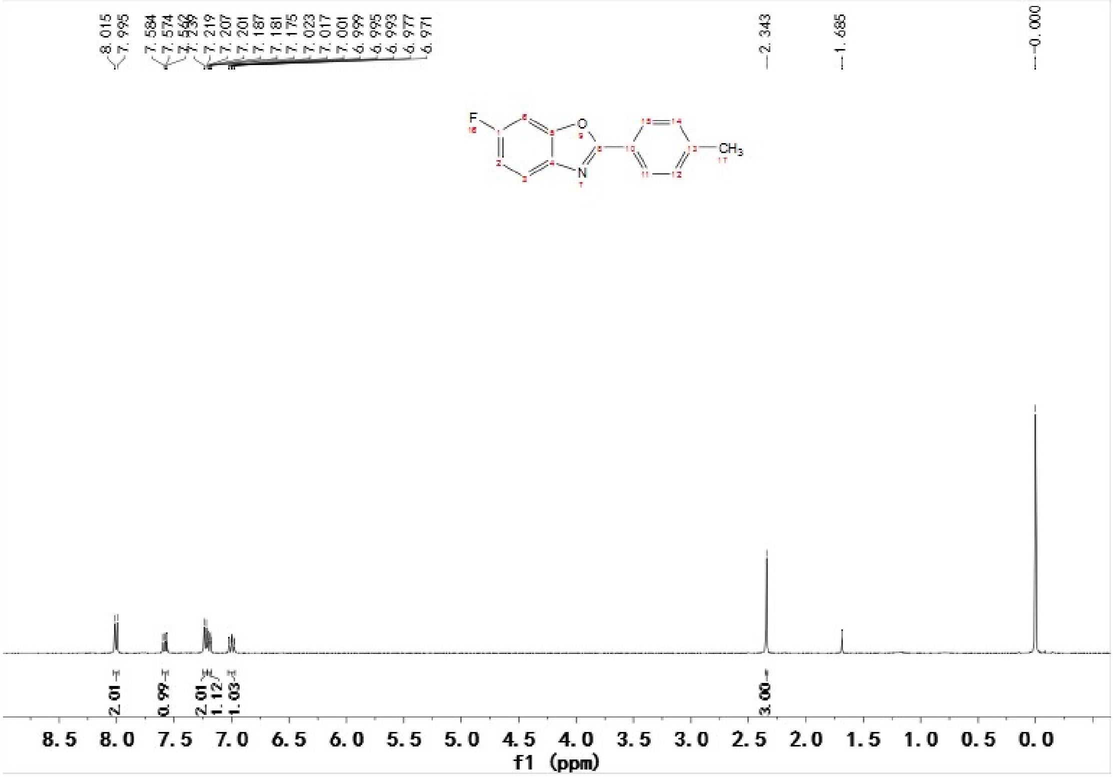
8.015
7.995
7.584
7.574
7.219
7.207
7.201
7.187
7.181
7.175
7.023
7.017
7.001
6.999
6.995
6.993
6.977
6.971
2.343
1.685
0.000
2.01
0.99
2.01
1.12
1.03
3.00
f1 (ppm)

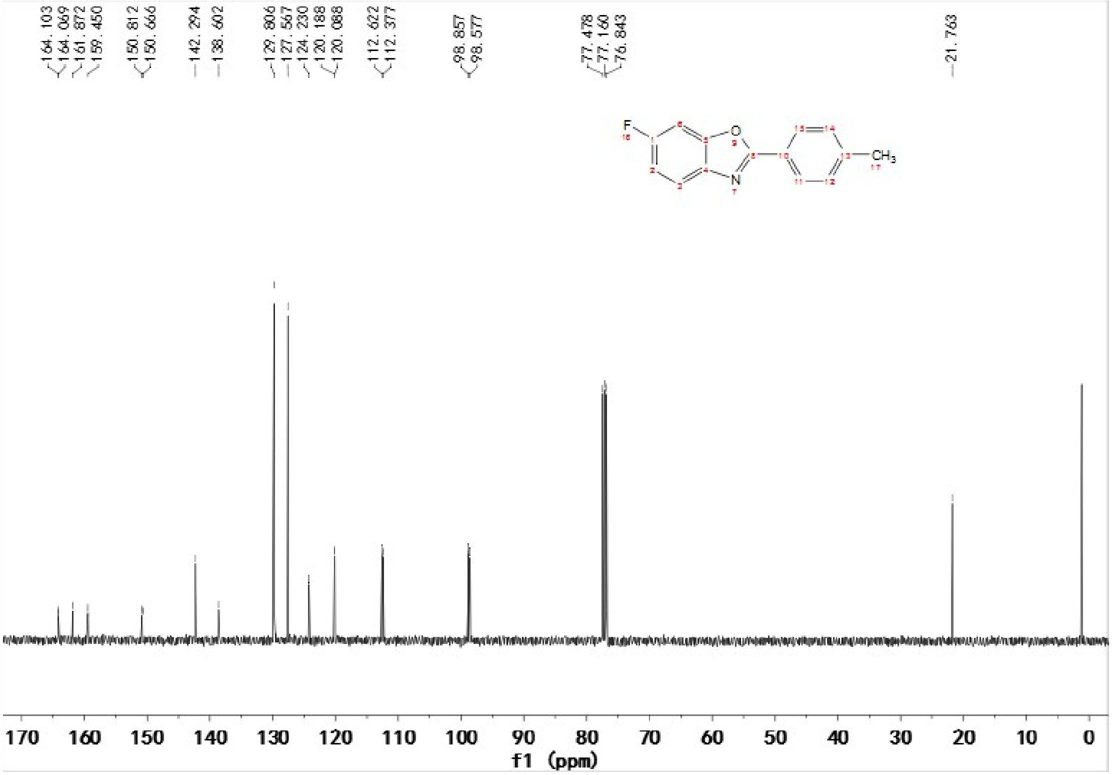
164.103
164.069
161.872
159.450
150.812
150.666
142.294
138.602
129.806
127.567
124.230
120.188
120.088
112.622
112.377
98.857
98.577
77.478
77.160
76.843
21.763
f1 (ppm)

3n

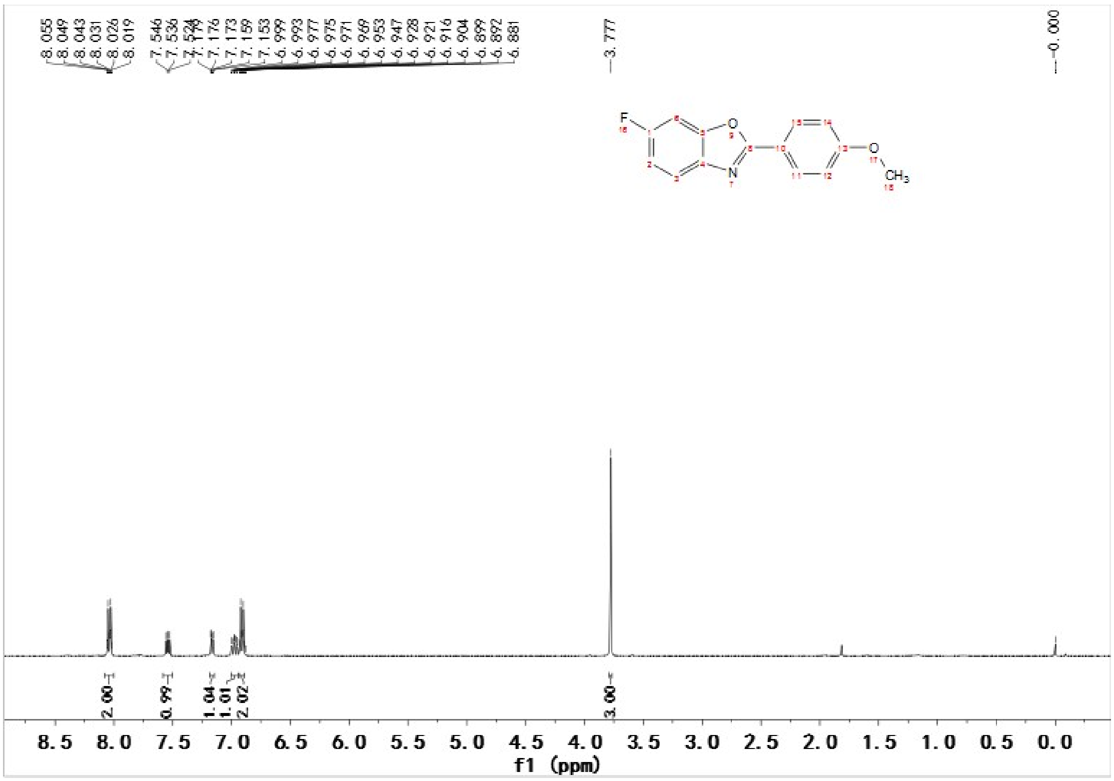
8.055
8.049
8.043
8.031
8.026
8.019
7.546
7.536
3.777
0.000
2.00
0.99
1.04
1.01
2.02
3.00
8.5 8.0 7.5 7.0 6.5 6.0 5.5 5.0 4.5 4.0 3.5 3.0 2.5 2.0 1.5 1.0 0.5 0.0
f1 (ppm)

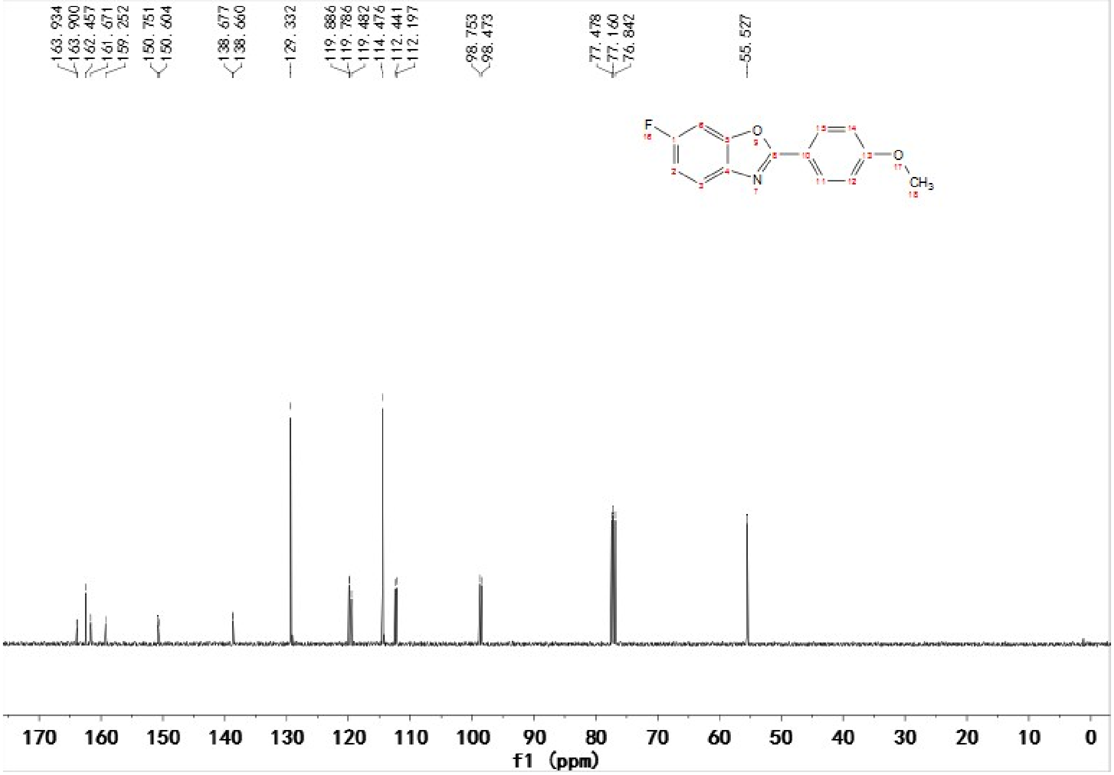
163.934
163.900
162.457
161.671
159.252
150.751
150.604
138.677
138.660
129.332
119.886
119.786
119.482
114.476
112.441
112.197
98.753
98.473
77.478
77.160
76.842
55.527
170 160 150 140 130 120 110 100 90 80 70 60 50 40 30 20 10 0
f1 (ppm)

3o

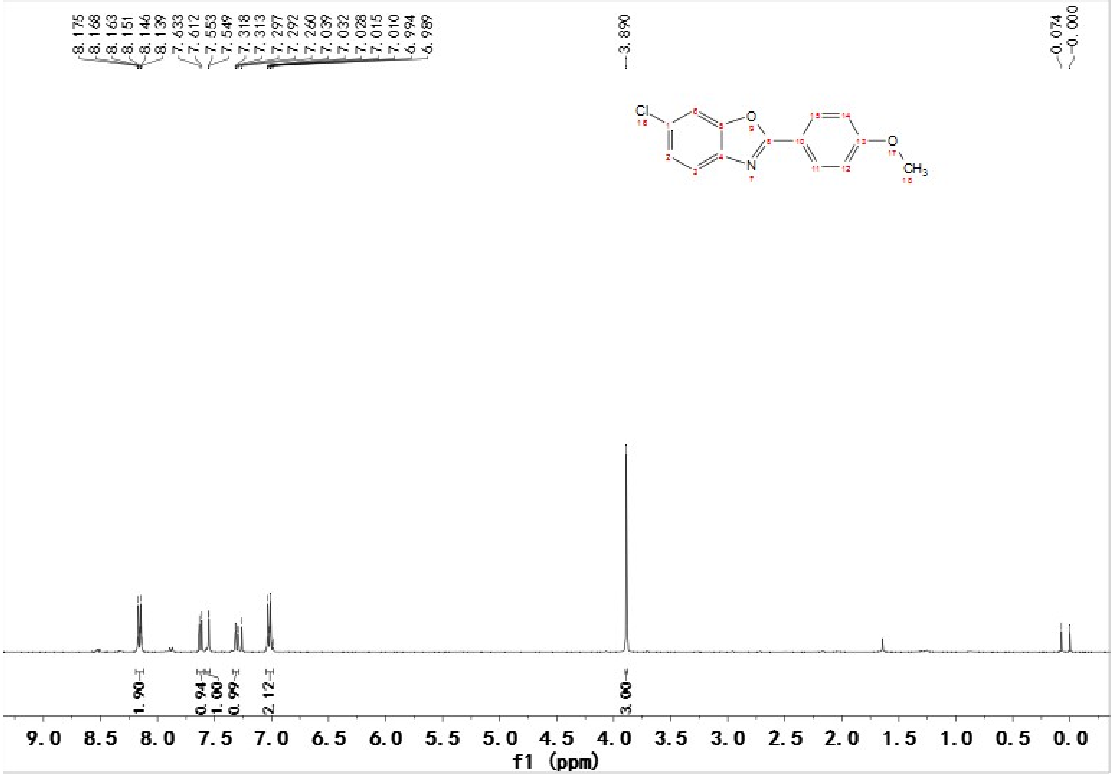

8.175
8.168
8.163
8.151
8.146
8.139
7.633
7.612
7.553
7.549
7.318
7.313
7.297
7.292
7.260
7.039
7.032
7.028
7.015
7.010
6.994
6.989
3.890
0.074
0.000
Cl
O
N
CH3
1.90
0.94
1.00
0.99
2.12
3.00
9.0 8.5 8.0 7.5 7.0 6.5 6.0 5.5 5.0 4.5 4.0 3.5 3.0 2.5 2.0 1.5 1.0 0.5 0.0
f1 (ppm)

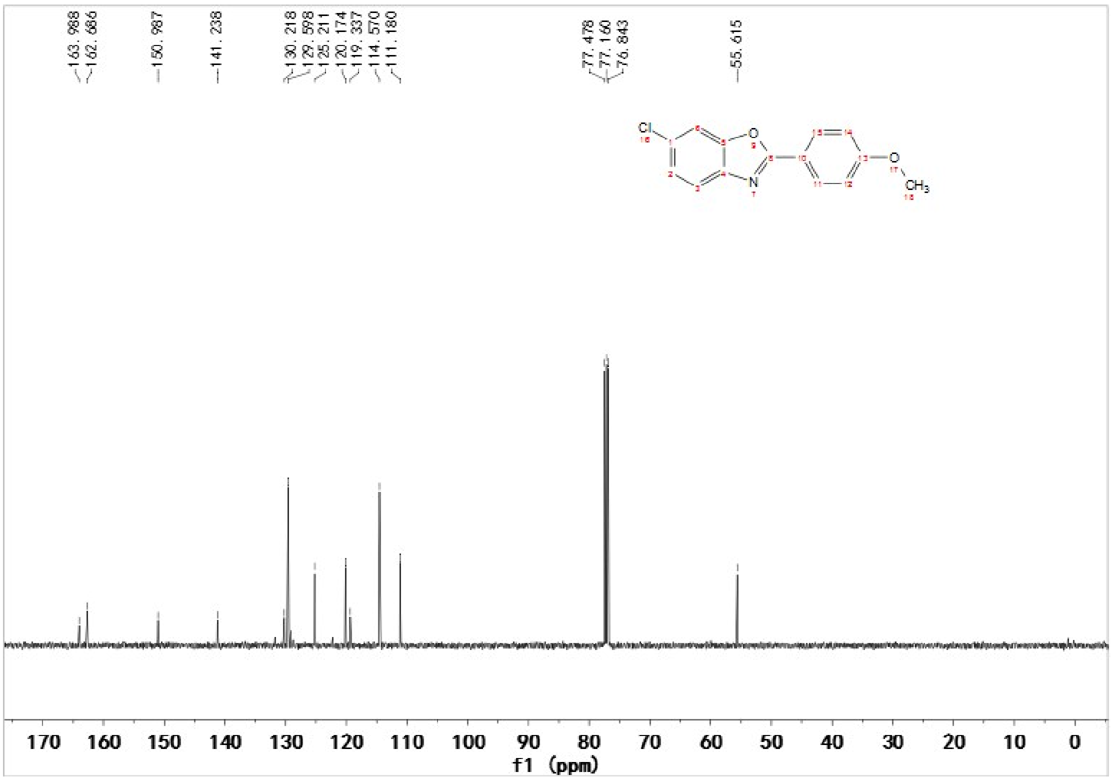

163.988
162.686
150.987
141.238
130.218
129.598
125.211
120.174
119.337
114.570
111.180
77.478
77.160
76.843
55.615
Cl
O
N
CH3
170 160 150 140 130 120 110 100 90 80 70 60 50 40 30 20 10 0
f1 (ppm)
